# Genome-wide identification and expression analysis of the calmodulin-binding transcription activator (CAMTA) family genes in tea plant

**DOI:** 10.1186/s12864-022-08894-x

**Published:** 2022-09-22

**Authors:** Bo Li, Shan He, Yiqian Zheng, Yu Wang, Xuxu Lang, Huan Wang, Kai Fan, Jianhui Hu, Zhaotang Ding, Wenjun Qian

**Affiliations:** 1 College of Horticulture, Qingdao Agricultural University, Qingdao, 266109 China; 2 Engineering Laboratory of Genetic Improvement of Horticultural Crops of Shandong Province, Qingdao, 266109 China

**Keywords:** *Camellia sisnensis*, Calmodulin-binding transcription activator, Tissue-specific analysis, Abiotic stresses, Hormones, Cold acclimation

## Abstract

**Background:**

As a type of calmodulin binding protein, CAMTAs are widely involved in vegetative and reproductive processes as well as various hormonal and stress responses in plants. To study the functions of *CAMTA* genes in tea plants, we investigated bioinformatics analysis and performed qRT-PCR analysis of the *CAMTA* gene family by using the genomes of ‘ShuChaZao’ tea plant cultivar.

**Results:**

In this study, 6 *CsCAMTAs* were identified from tea plant genome. Bioinformatics analysis results showed that all CsCAMTAs contained six highly conserved functional domains. Tissue-specific analysis results found that CsCAMTAs played great roles in mediating tea plant aging and flowering periods. Under hormone and abiotic stress conditions, most *CsCAMTAs* were upregulated at different time points under different treatment conditions. In addition, the expression levels of *CsCAMTA1*/*3*/*4*/*6* were higher in cold-resistant cultivar ‘LongJing43’ than in the cold-susceptible cultivar ‘DaMianBai’ at cold acclimation stage, while *CsCAMTA2*/*5* showed higher expression levels in ‘DaMianBai’ than in ‘LongJing43’ during entire cold acclimation periods.

**Conclusions:**

In brief, the present results revealed that CsCAMTAs played great roles in tea plant growth, development and stress responses, which laid the foundation for deeply exploring their molecular regulation mechanisms.

**Supplementary Information:**

The online version contains supplementary material available at 10.1186/s12864-022-08894-x.

## Introduction

The divalent ions of calcium (Ca^2+^) is an universal secondary messenger, which served as a core sensor and regulator of plants in dealing with growth, development and various environment stimuli [1–4]. Until now, there are three important calcium sensors, including calmodulins/calmodulin-like proteins (CaMs/CMLs), calcineurin B-like proteins (CBLs) and calcium-dependent protein kinases (CDPKs) have been identified in plants [5]. Among them, CaMs are regarded as main calcium sensors in the process of calcium signal transduction, which can sense the change of calcium concentration and participate in numerous of physiological activities by regulating downstream target proteins in plant. It has been demonstrated that more than 90 types of transcription factors, including CAMTAs (CaM-binding transcription activators), bZIPs (basic leucine zipper), MYBs (myeloblastosis), NACs (NAM/ATAF/CUC) and WRKYs (WRKYGQK), etc., were reported as downstream target TFs that regulated by CaMs [6–9]. Among them, CAMTAs, also called signal responsive (SR) proteins or ethylene-induced CaM-binding proteins (EICBP), are referred as central CaM-binding proteins (CBPs), which have been confirmed to mediate entire life cycles of multicellular eukaryotes from plants to humans [10–12]. In plant, it has been clear that CAMTAs contain six conserved functional domains, including nuclear localization signals (NLS) function in targeting protein into nucleus, CG-1 domain (CG-1) implicated in DNA binding [11, 12], TIG domain implicated in nonspecific DNA interactions [13], ankyrin (ANK) repeats involved in protein–protein interaction [14, 15], IQ motifs (IQXXXRGXXXR) combined with CaM [16], and calcium dependent CaM binding domain(CaMBD)contributed to the combination of Ca^2+^-loaded CaM to CAMTAs [17]. In addition to functional domains, two *cis*-acting elements, (G/A/C) CGCG (C/G/T) and (A/C) CGTGT, have been identified as specific CAMTA-binding sites in plants [12, 17, 18].

Currently, lots of *CAMTAs* genes have been identified from different plant species, such as 6 *AtCAMTAs* from Arabidopsis [11], 10 *VvCAMTAs* from grape [19], 9 *ZmCAMTAs* from maize [18], 7 *SlCAMTAs* from tomato [20], 5 *MaCAMTAs* from banana [21], 7 *MsCAMTAs* from alfalfa [22], 9 *LuCAMTAs* from flax [23], 9 *CsCAMTAs* from citrus [24]. Among them, numerous CAMTAs have been shown to play great roles in the regulation of plant growth and development, hormones, biotic and abiotic stress responses, especially in low temperature responses [12, 15, 25, 26]. Under cold condition, the increased Ca^2+^ contents could promote the combination of CAMTAs with ‘CCGAC’ *cis*-acting element, and then induce the expressions of many downstream genes, thus rapidly respond to cold stress and enhance cold adaptability and tolerance of plants [24, 27]. In *Arabidopsis*, the spatio-temporal expressions of all 6 *AtCAMTAs* were rapidly and differentially influenced by various hormones, biotic and abiotic stresses [3]. Galon et al. (2010) reported that *AtCAMTA1-3* were referred as negative regulators of auxin, which correlated to red light and high light responses, while *AtCAMTA4-6* were functioned as positive regulators to regulate auxin signaling and homeostasis [28]. Besides, *AtCAMTA1* transcripts were triggered by exogenous auxin with a cell-specific manner, mutation of *AtCAMTA1* stunted root and rosette leaves development, meanwhile, *camta1* showed higher sensitivity to drought stress with poor water use efficiency (WUE), low photosystem II efficiency, declined in relative water content (RWC) and reduced survivability [29]. As a negative regulator of plant immunity, AtCAMTA3 could inhibit *enhanced disease susceptibility 1* (*EDS1*) transcripts by interacting with its promoter, while the mutation of *AtCAMTA3* could stimulate *EDS1* transcripts and improve salicylic acid accumulation, and thus enhance disease resistance of *camta3* mutants [23]. Even so, it has also reported that *AtCAMTA3* could positively mediate the freezing tolerance of *Arabidopsis* through binding to the CG-1 DNA-binding sites in the promoters of *core binding factor* (*CBFs*) [30]. Further research found that CAMTA1-3 could synergistically induce the highest expressions of *CBF1*-*3* after 2 h of 4 °C chilling treatment, following lead to the up-regulation of more than 15% cold responsive genes in CBF independent pathway, and thus enhance the freezing tolerance of *Arabidopsis* [31]. Apart from 6 *AtCAMTAs*, the functions of many *CAMTAs* in plants also have been extensively explored. In citrus, the expressions of 8 *CitCAMTAs* genes were regulated by various stress and hormone treatments [24]. 7 *SlSR*/*CAMTAs* of tomato showed differential expressions during fruit development and ripening [20]. In wheat genome, about 584 genes were predicted to contain ACGCGG/CCGCGT *cis*-acting elements in their promoter regions, suggesting that these genes could be considered as potential target genes of TaCAMTAs, which mainly participated in RNA regulation, protein degradation, signaling transduction, biotic and abiotic stresses, hormone metabolism, and lipid metabolism [25]. Similarly, many stress-related *cis*-acting elements also presented in the promoter regions of some *ZmCAMTA* genes, suggesting that *ZmCAMTAs* widely involved in stress responses. Specifically, *ZmCAMTAs* transcripts were rapidly triggered by maize rough dwarf disease (RBSDV) infection, of which *ZmCAMTA6*/*7a* showed differential expressions between disease-tolerant and disease-sensitive cultivars [18].

Tea plant (*Camellia sinensis*) is a type of evergreen woody plants, which is mainly distributed in tropical and subtropical regions of the Northern hemisphere. Generally, tea plant is suitable for acid soil (pH4.5–6.5), high moisture, and normal temperature conditions. However, with the frequent occurrence of extreme climates, such as freezing, cold spell in spring, drought and heat etc., the growth, tea production and quality are seriously retarded in recent years. Therefore, more and more researchers are focusing on how to improve the stress-resistance of tea plants, of which the molecular mechanisms in responding to environmental stimuli are the main research areas. Currently, lots of studies have demonstrated that calcium signaling plays critical role in dealing with various stresses in tea plant, and multiples genes (e.g. *CsCBLs, CsCDPKs*, *CsCIPKs* and *CsCMLs)* involved in calcium signaling *were* up-regulated under stress conditions [26, 32]. Based on the tea plant genome, many genes associated with calcium signaling perception and transduction have been comprehensively identified and further performed expression analysis under various stresses treatment conditions [32–34]. However, as the central CBPs in calcium signaling pathway, the functions of CAMTAs have not been extensively explored in tea plant. In the present study, we systematically performed genome-wide analysis of *CAMTA* genes and widely explored their tissue-specific and spatial–temporal expressions profiles in tea plant. These results will provide a solid theoretical foundation for intensive study on the role of calcium signal in stress responses of tea plant.

## Methods

### Plant materials and stress treatments

The one bud and two leaves, mature leaves, senescent leaves, flower buds, mature flowers, young fruits, young stems, mature stems and roots of ten-year-old clonal tea plant cultivar ‘ShuChaZao’ were sampled for tissue-specific analysis. Each tissue was performed three independent biological replicates, and all samples were quickly frozen in liquid nitrogen and stored at -80 °C until used.

The one-year-old clonal cuttings of ‘ShuChaZao’ were used to perform 3% H_2_O_2_ treatment. Before processing, all cuttings were moved into chamber for adjusting growth one week, and the culture conditions were as follows: temperature 25℃, 14 h light/10 h darkness, humidity 75%. For H_2_O_2_ treatments, the tea plants were sprayed with 3% H_2_O_2_, and the samples were collected at 0 h, 6 h, 12 h and 24 h. Three biological replicates were performed for each treatment, and all samples were frozen in liquid nitrogen and stored at -80 °C. The above mentioned tea plants were cultivated in the greenhouse of the Tea Research Institute of Qingdao Agricultural University (TRI, QAU, N36°33′, E120°4′).

The methods of cold, polyethylene glycol (PEG), NaCl, abscisic acid (ABA) and gibberellin (GA) treatments were performed as described by Wang et al. (2021) [35]. In brief, one-year-old clonal cutting seedlings of the ‘LongJing43’ cultivar with similar growth potential were used to process different treatments. 4 °C, PEG-6000 (10% (w/v)) and 250 mmol·L^−1^ NaCl were respectively used to imitate cold (CT), drought (DT) and salt (ST) treatment, and 100 μmol·L^−1^ ABA and 100 μmol·L^−1^ GA were sprayed onto the surfaces of tea leaves to imitate hormone treatments. Each treatment was proceeded 2 d and three independent biological replicates. The third and/or fourth mature leaves from the terminal bud were sampled at 0, 12, 24 and 48 h within treatment periods, and then all samples were quickly frozen in liquid nitrogen and stored at -80 °C until used. The above mentioned tea plants were cultivated in the greenhouse of the Tea Research Institute of Qingdao Agricultural University (TRI, QAU, N36°33′, E120°4′).

Eighteen-year-old of two tea plant cultivars, ‘LongJing43’ and ‘DaMianBai’, with different cold resistance as reported by Wang et al. (2019) [36], were used to perform cold acclimation (CA) analysis. The sampling method was performed as described by Qian et al. (2018) [37]. The above mentioned tea plants were cultivated at the Tea Research Institute of the Chinese Academy of Agricultural Sciences (TRI, CAAS, N30°10′, E120°5′).

### Genome-wide identification of the *CAMTA* genes from tea plant genome

In order to obtain putative *CAMTA* genes, four Hidden Markov Models (HMM) files of CAMTA functional domains, including CG-1 domain (PF03859), IPT/TIG domain (PF01833), Ankyrin repeat (PF00023), and IQ domain (PF00612) were respectively downloaded from protein families (Pfam) database (http://pfam.xfam.org/) [38]. Subsequently, the HMM profiles were respectively performed blast search in the tea plant protein database of the ‘ShuChaZao’ cultivar as reported by Wei et al. (2018) by using HMMER 3.0 software [39]. Following, both the simple modular architecture research tool (SMART) server (http://smart.embl-heidelberg.de/) [40] and conserved domain database of national center for biotechnology information (NCBI) (https://www.ncbi.nlm.nih.gov/cdd/advanced) [41] were used to further ensure whether the obtained sequences contain the conserved CAMTA functional domains, such as the CG-1 domain, IQ motifs, Ank repeats, and IPT/TIG. Finally, those sequences that met the above conditions were reserved for subsequent analysis.

### Bioinformatics analysis of CsCAMTAs in tea plant

The opening reading frame (ORF) lengths of *CsCAMTAs* were predicted by using the NCBI ORF finder website (https://www.ncbi.nlm.nih.gov/orffinder/). The molecular weights, theoretical pI, instability index and aliphatic index were predicted by using protein parameter (ProtParam) tool (http://web.expasy.org/protparam/) [42]. Signal peptides and transmembrane regions (TMHs) were respectively predicted with the Signal peptide (SignalP) server (http://www.cbs.dtu.dk/services/SignalP) [43] and the transmembrane protein topology with a hidden Markov model (TMHMM) Server v.2.0 (http://www.cbs.dtu.dk/services/TMHMM/) [44], and the plant multiple protein locations (PlantmPLoc) web server (http://www.csbio.sjtu.edu.cn/bioinf/plant-multi/) [45] was used to predict the sub-cellular location of CsCAMTAs.

### Phylogenetic analysis of CsCAMTAs

In order to explore the evolutionary relationship of CAMTAs in different plant species, a total of 88 CAMTAs protein sequences (Table S1) originated from tea plant, *Arabidopsis*, *Oryza sativa*, *Populus trichocarpa*, *Malus pumila*, *Triticum aestivum*, *Nicotiana tabacum* and *Zea mays* were used to construct a phylogenetic tree based on the neighbor-joining method of MEGA 7.0 software [46]. The detailed parameters were as follows: 1000 repeated bootstrap tests, *p*-distance method and pairwise deletion treatment. Finally, the ITOL web server (https://itol.embl.de/) [47] was further used to beautify and generate the phylogenetic tree.

### Chromosomal distribution, Ka/Ks ratios, and synteny analysis of *CsCAMTAs*

The chromosomal positions of *CsCAMTAs*, collinearity analysis within ‘ShuChaZao’ genome and the synteny analysis of ‘ShuChaZao’ cultivar associated with *Arabidopsis*, *Oryza sativa*, *Zea may* and another two tea plant cultivars (‘HuangDan’ and ‘TieGuanYin’) genomes were performed and visualized by using TBtools software as demonstrated by Chen et al. (2020) [48]. Besides, the synonymous substitution rate (Ks) values, nonsynonymous substitution rate (Ka) values and the ratios of Ka/Ks were also performed by using TBtools software [48]. The genomes of *Arabidopsis*, *Oryza sativa*, and *Zea may* were downloaded from NCBI web (https://www.ncbi.nlm.nih.gov/datasets/genomes/). The genomes of ‘HuangDan’ and ‘TieGuanYin’ cultivars were downloaded from national genomics data center (NGDC) (https://ngdc.cncb.ac.cn/) [49] by using accession number GWHAZTZ00000000 [50] and GWHASIV00000000 [51].

### Gene structure, protein domain distribution and *cis*-acting element analysis

The coding sequences (CDSs) and the corresponding genomic sequences of *CsCAMTAs* were submitted into the gene structure display server 2.0 (GSDS2.0) website (http://gsds.cbi.pku.edu.cn/) [52] to predict their exon–intron structures. The SMART web server (http://smart.embl-heidelberg.de/) [40], classification of protein families (InterPro) database (https://www.ebi.ac.uk/interpro/search/sequence/) [53], Motif Scan database (https://myhits.sib.swiss/cgi-bin/motif_scan#GRAPHIC) [54] and CaMBD database (http://calcium.uhnres.utoronto.ca/ctdb/ctdb/home.html) was used to search the putative functional domains of CsCAMTAs. In order to understand the expression regulation factors of *CsCAMTAs*, 2000-bp upstream noncoding region sequence of the translation initiation site (ATG) in each *CsCAMTA* genome sequence was submitted into plant *cis*-acting regulatory element (PlantCARE) web server (http://bioinformatics.psb.ugent.be/webtools/plantcare/html/) [55] to predict putative *cis*-acting elements involved in responding to stresses and hormones. The results were further visualized in illustrator for the presentation and visualization of biological sequences (IBS) web server (http://ibs.biocuckoo.org/online.php) [56].

### qRT-PCR analysis

RNA extraction kit (Bioflux, Hangzhou, China) and RT reagent Kit (Takara, Dalian, China) were respectively used to isolate total RNA and synthesize the first-strand cDNA following the corresponding instruction of kits. The qRT-PCR technique was performed as described by Wang et al. (2021) [35]. In brief, total of 20.0 μL reaction mix (10.0 μL SYBR Premix Ex Taq, 2 μL cDNA, 1.6 μL forward/reverse primers, and 6.4 μL distilled water) were amplified according to the following qRT-PCR programs: 95 °C, 15 s (step 1); 94 °C, 5 s following 60 °C, 30 s with 40 cycles (step 2); adding melting curve (step 3). A reference gene, *polypyrimidine tract-binding protein* (*CsPTB*) of tea plant [57] was used to quantify the relative expression levels of *CsCAMTAs*. The results were calculated by 2^−ΔCt^ or 2^−ΔΔCt^ method [58], and finally visualized as the mean values ± standard error (± SE). The qRT-PCR primers are listed in Table S2.

## Results

### Identification of *CAMTA* family genes in tea plants

Based on HMM files of CAMTA functional domains, and the conformation of the SMART server and the CD databases of NCBI, total of six putative *CsCAMTAs*, named as *CsCAMTA1*-*6*, were identified from ‘ShuChaZao’ tea plant cultivar genome. As bioinformatics analysis results showed that the ORF lengths of *CsCAMTAs* were varied from 2.772 kb to 3.294 kb, the amino acids lengths ranged from 924 to 1098 aa, the molecular weights (MW) range of 104.48–123.95 kDa, the theoretical isoelectric point (pIs) changed from 5.39 to 7.38, and all of them were predicted to be unstable proteins except for CsCAMTA4. Besides, all of them were predicted to contain no signal peptides and TMHs, and predicted to locate in nucleus (Table 1).

**Table 1:** Basic information of CsCAMTAs. ORF, Opening reading fame; AA, The numbers of amino acid residues; MW, Molecule weight; pI, Theoretical isoelectric point; Loc, Subcellular location; SignalP, Signal peptide; TMHs, Transmembrane helices

Gene name	Accession number	ORF (bp)	AA	MW (kDa)	pI	Instability index	Aliphatic index	Loc	SignalP	TMHs
CsCAMTA1	XP_028075461.1	2829	943	106.53	6.67	unstable	76.35	Nucleus	NO	NO
CsCAMTA2	XP_028093707.1	3024	1008	112.71	5.88	unstable	74.42	Nucleus	NO	NO
CsCAMTA3	XP_028051249.1	2988	996	111.12	5.79	unstable	79.24	Nucleus	NO	NO
CsCAMTA4	XP_028068006.1	2931	977	108.78	7.38	stable	78.22	Nucleus	NO	NO
CsCAMTA5	XP_028069892.1	3294	1098	123.95	5.39	unstable	75.14	Nucleus	NO	NO
CsCAMTA6	XP_028094568.1	2772	924	104.48	6.43	unstable	76.04	Nucleus	NO	NO

### Phylogenetic analysis of CsCAMTAs

**Fig. 1:**
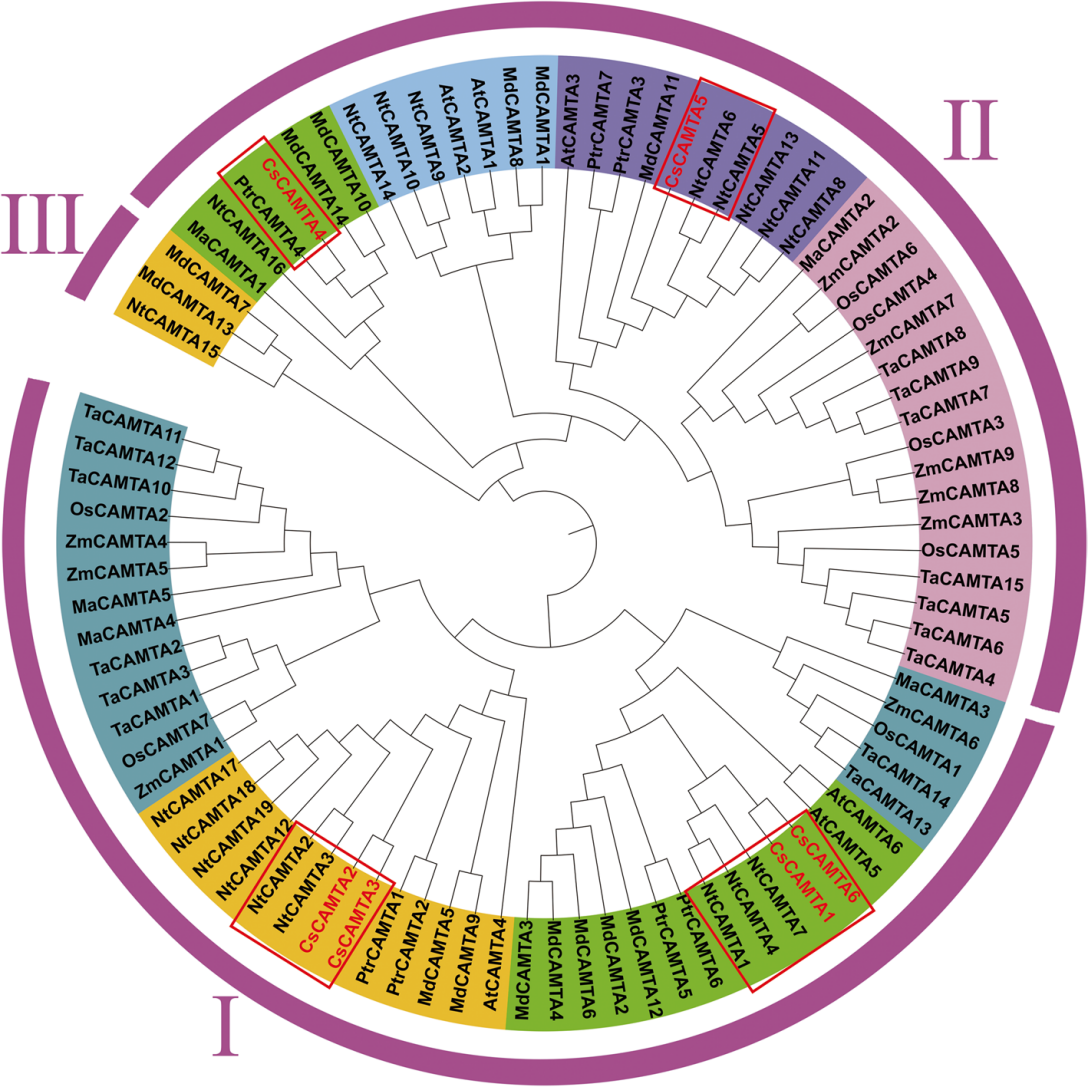
Phylogenetic analysis of CsCAMTAs and known CAMTAs of other plant species. A total of 88 CAMTA protein sequences from tea plant, *Arabidopsis*, rice, banana, poplar, apple, wheat, tobacco, and maize were used to construct phylogenetic tree. The amino acids sequences were listed in Table S1

Phylogenetic analysis result showed that all 88 CAMTAs were grouped into three subfamilies, of which 4 CsCAMTAs were clustered into subfamily I (Fig. 1). Besides, 6 CsCAMTAs were clustered into 3 subgroups and showed closest relationship with NtCAMTAs except for CsCAMTA4. One 2:2 ortholog gene-pairs (CsCAMTA2 and CsCAMTA3/NtCAMTA2 and NtCAMTA3) with more than 88% bootstrap values were found between tea plant and tobacco. Furthermore, the 2:3 ortholog gene pairs (CsCAMTA1 and CsCAMTA6/NtCAMTA1, NtCAMTA4 and NtCAMTA7) with more than 92% bootstrap values were identified between tea plant and tobacco.

### Chromosomal distribution and synteny analysis of *CsCAMTAs*

**Fig. 2:**
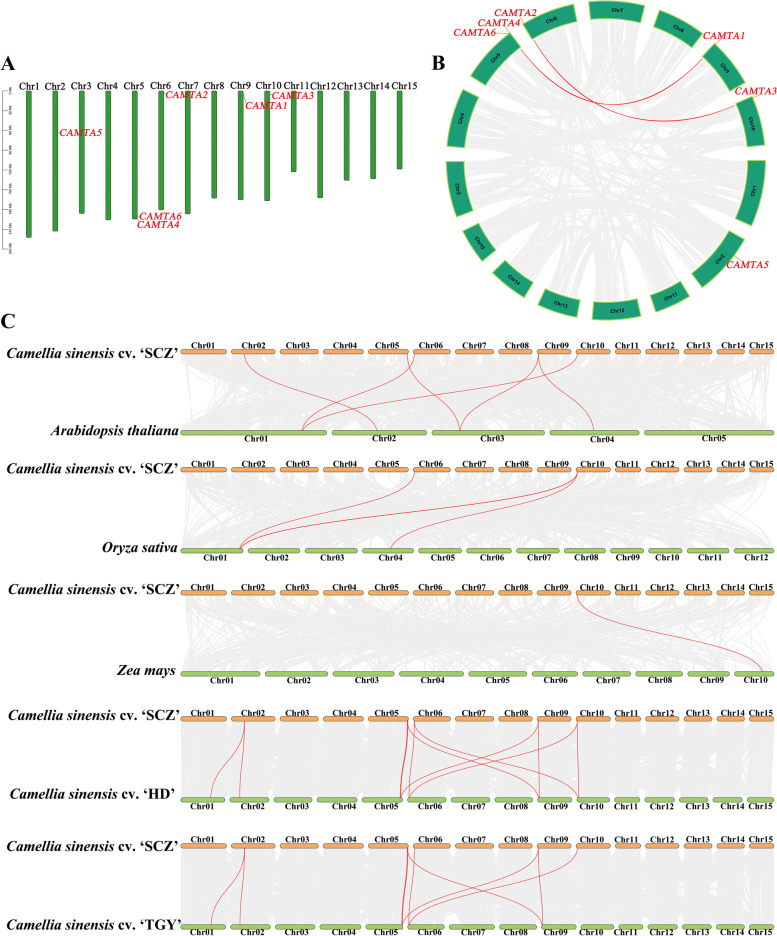
Chromosomal distribution and synteny analysis of *CsCAMTAs*. **A** The chromosomal distribution of *CsCAMTAs* in ‘ShuChaZao’ genome. **B** Collinearity analysis of *CsCAMTAs* within ‘ShuChaZao’ genome. **C** The interspecies synteny analysis of *CsCAMTAs* in ‘ShuChaZao’ associated with *Arabidopsis*, *Oryza sativa*, *Zea mays*, and ‘HuangDan’ and ‘TieGuanYin’ cultivars

To investigate the chromosomal distribution of *CsCAMTAs*, the CDS sequences of *CsCAMTAs* were matched on ‘ShuChaZao’ genome by means of TBtools software. As shown in Fig. 2A and Table S3, *CsCAMTA1*-*3*, *5* were respectively distributed on Chr9, Chr6, Chr10, and Chr2, while *CsCAMTA4*/*6* were co-distributed on Chr5. The similar distributions were also respectively found in the genomes of ‘HuangDan’ and ‘TieGuanYin’ cultivars with the exception of *CsCAMTA5* (Fig. S1).

In addition to chromosomal distribution, we also explored the collinearity relationships of *CsCAMTAs* within the genome of ‘ShuChaZao’ cultivar. As Fig. 2B and Table S4 showed that a same chromosomal distribution result was also obtained by collinearity analysis in the genome of ‘ShuChaZao’ cultivar. Besides, two segmental duplication events (*CsCAMTA1*/*6* and *CsCAMTA2*/*3*) were identified in the ‘ShuChaZao’ tea plant cultivar genome with the exception of *CsCAMTA4*/*5*. On the other hand, the Ka/Ks ratios of these *CsCAMTAs* were calculated, and we found that the Ka/Ks ratios of 17 pairs of *CsCAMTAs* were all lower than 1 (Table S5), which suggested that all *CsCAMTAs* underwent purification selection during evolution periods.

To further insight into the evolutionary relationships of *CsCAMTAs*, five comparative syntenic maps of ‘ShuChaZao’ tea plant cultivar genome associated with *Arabidopsis* genome, *Oryza sativa* genome, *Zea may* genome and another two tea plant cultivar genomes (‘HuangDan’ and ‘TieGuanYin’) were constructed respectively. As shown in Fig. 2C and Table S6, both *CsCAMTA2* and *CsCAMTA3* possess common orthologous genes in *Arabidopsis* (*AtCAMTA4*, AT1G67310), rice (*OsCAMTA7*, LOC4327253), ‘HuangDan’ (HD.03G0000940.t1 and HD.11G0000910.t1) and ‘TieGuanYin’ (TGY050530.t1 and TGY050530.t1). Besides, *CsCAMTA3* possesses another orthologous gene in rice (*OsCAMTA2*, LOC4335664) and *Zea mays* (ZmCAMTA4, LOC103642708). *CsCAMTA4* possesses one orthologous gene in ‘HuangDan’ (HD.08G0000210.t1) and ‘TieGuanYin’ (TGY050137.t1), respectively. *CsCAMTA5* possesses one orthologous gene in *Arabidopsis* (*AtCAMTA3*, AT2G22300), and two orthologous genes in ‘HuangDan’ (HD.01G0023840.t1 and HD.04G0022130.t1) and ‘TieGuanYin’ (TGY007127.t1 and TGY014016.t1) respectively. Similarly, both *CsCAMTA1* and *CsCAMTA6* also possess one common orthologous gene in *Arabidopsis* (*AtCAMTA6*, AT3G16940), and two orthologous genes in ‘HuangDan’ (HD.08G0000960.t1 and HD.10G0029650.t1) and ‘TieGuanYin’ (TGY049973.t1 and TGY077391.t1) respectively. Moreover, *CsCAMTA1* possesses another orthologous gene in *Arabidopsis* (*AtCAMTA5*, AT4G16150). These results were corresponded to the phylogenetic analysis result, where we found the orthologous gene pairs were grouped into same branches.

### *Cis*-acting elements and exon–intron structures analysis of *CsCAMTAs*

**Fig. 3:**
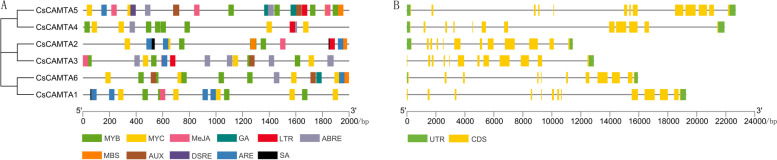
The *cis*-acting elements in promoters of *CsCAMTAs* and exon–intron structures of *CsCAMTAs*. **A** *cis*-acting elements in promoters of *CsCAMTAs*. 2000-bp upstream noncoding region sequences of *CsCAMTAs* were used to predict cis-acting elements, and different colored blocks represent different elements. **B** The exon–intron structures of *CsCAMTAs*. Green boxes represent untranslated upstream/downstream regions, yellow boxes represent exons, and lines indicate introns

To investigate the regulatory mechanisms of *CsCAMTAs* in response to various stresses and hormones, the *cis*-acting elements in 2000 bp promoter sequence of each *CsCAMTAs* were predicted. As shown in Fig. 3A, the distribution, number and type of *cis*-acting elements of *CsCAMTAs* are varied among each other, and many myeloblastosis (MYB) and myelocytomatosis (MYC) binding sites contained in the promoter region of each *CsCAMTA*. Besides, *CsCAMTAs* possess many light response *cis*-acting elements in their promoter regions. In addition, many stress-responsive elements, such as low-temperature responsiveness element (LTR), abscisic acid responsiveness element (ABRE), anaerobic induction element (ARE), salicylic acid responsiveness element (SA), MYB binding site involved in drought-inducibility element (MBS), methyl jasmonate responsiveness element (MeJA), auxin responsiveness element (AUX), defense and stress responsiveness element (DSRE) were enriched in the promoters of *CsCAMTAs*. For example, LTR elements were enriched in the promoter regions of *CsCAMTA2*-*5*, ABRE elements were enriched in the promoter regions of *CsCAMTA3*-*6*, MBS elements were enriched in the promoter regions of *CsCAMTA2*/*5*/*6*. These results demonstrated that each *CsCAMTA* plays an important role in coping with diurnal changes, hormones, and abiotic stresses.

GSDS 2.0 was used to explore the structural diversity of *CsCAMTAs*. As Fig. 3B shown, the exon–intron distribution patterns of *CsCAMTAs* gene family are varied in terms of intron length and exon number. Among them, *CsCAMTA2*/*3*/*4* possess same numbers of exons and introns, including 12 exons and 11 introns. Besides, both *CsCAMTA1* and *CsCAMTA5* contain 13 exons and 12 introns, while *CsCAMTA6* contains 11 exons and 10 introns.

### Motifs and protein domain compositions of CAMTAs

**Fig. 4:**
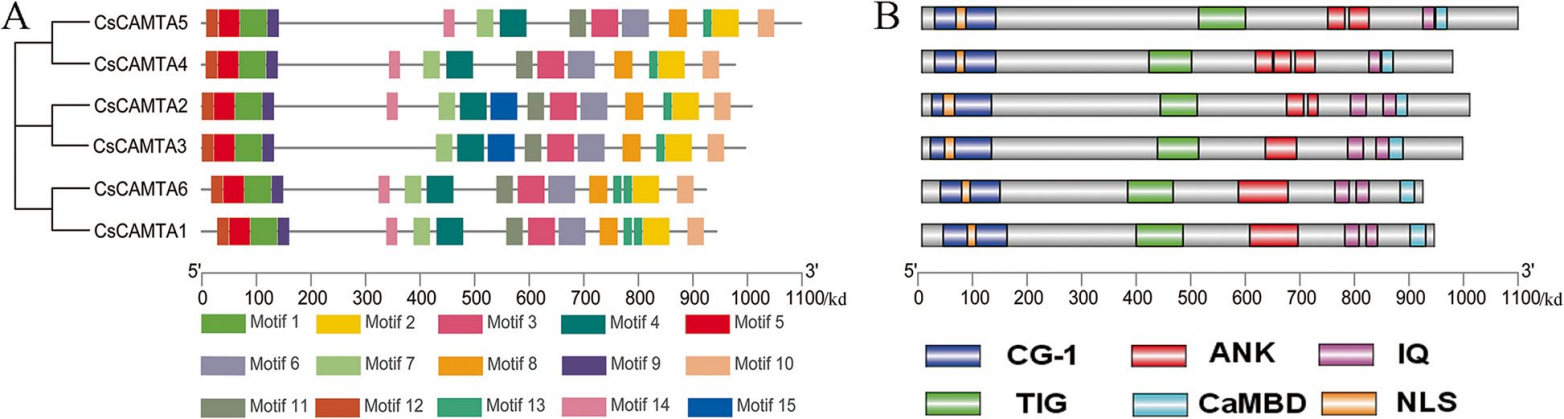
Motifs and Conserved protein domains of CAMTAs. **A** Conserved motifs of CAMTAs. Different motifs were showed with different colored squares. **B** Conserved domains of CsCAMTAs. Different domains were showed with different shapes

MEME tool was used to comprehend the motif conserveness among all 6 CsCAMTAs. Correspondingly, CsCAMTAs found to be highly conserved, and all of them contain motif 1–12 (Fig. 4A). However, motif 14 is not contained in CsCAMTA3, and motif 15 is just contained in CsCAMTA2/3. In addition, both CsCAMTA1 and CsCAMTA6 contain 2 motif 13, while CsCAMTA2/3/4/5 only contain 1 motif 13 respectively.

To further dissect the functions of CsCAMTAs, the conserved domains of each CsCAMTA were analyzed by the SMART server and the CD databases of NCBI. As shown in Fig. 4B, all of CsCAMTAs possess 6 conserved domains, including NLS, CG-1, ANK repeats, IQ motifs, TIG and CaMBD domain (Fig. 4B). However, the numbers of IQ motifs and ANK repeats were varied from each other, among of which CsCAMTA1/2/3/6 contain 2 IQ motifs respectively, and CsCAMTA4/5 just contain 1 IQ motif. Besides, 3 ANK repeats were contained in CsCAMTA4, 2 ANK repeats were contained in CsCAMTA2/5, and 1 ANK repeat was contained in CsCAMTA1/3/6 respectively. Moreover, we found the conserved CG-1 located in motif 1/9/12, and TIG, CaMBD and NLS domains respectively located in motif 4/10/5 were all contained in CsCAMTAs. These results demonstrated that each of CsCAMTA may be targeted by different CaMs or served as different types of binding proteins.

### Tissue-specific analysis of *CsCAMTAs* in different tea plant tissues

**Fig. 5:**
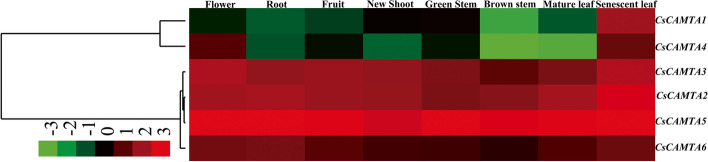
Tissue-specific expression patterns of *CsCAMTAs* in different tea plant tissues. The data were calculated with 2^–ΔCt^ method, and the red and green colors represent higher and lower expression levels respectively in the heat map. The colorbar was displayed on the lower-left of the heat map

The tissue-specific of *CsCAMTAs* were detected in 9 different tissues of ‘ShuChaZao’ cultivar. As Fig. 5 shown, the transcription abundance of each *CsCAMTA* was varied across the various tissues. Among them, *CsCAMTA2*/*3*/*5*/*6* showed high transcription abundances in all detected tea plant tissues, and the transcription abundances of these genes were higher in senescent leaves, flowers and roots than that in the other tea plant tissues. Similarly, *CsCAMTA1*/*4* also showed higher expression levels in senescent leaves and flower than that in the other tea plant tissues. In brief, our results demonstrated that *CsCAMTAs* mediated entire vegetative and reproductive progress of tea plant, especially in aging and flowering periods.

### Expression analysis of *CsCAMTAs* under various abiotic stress conditions

**Fig. 6:**
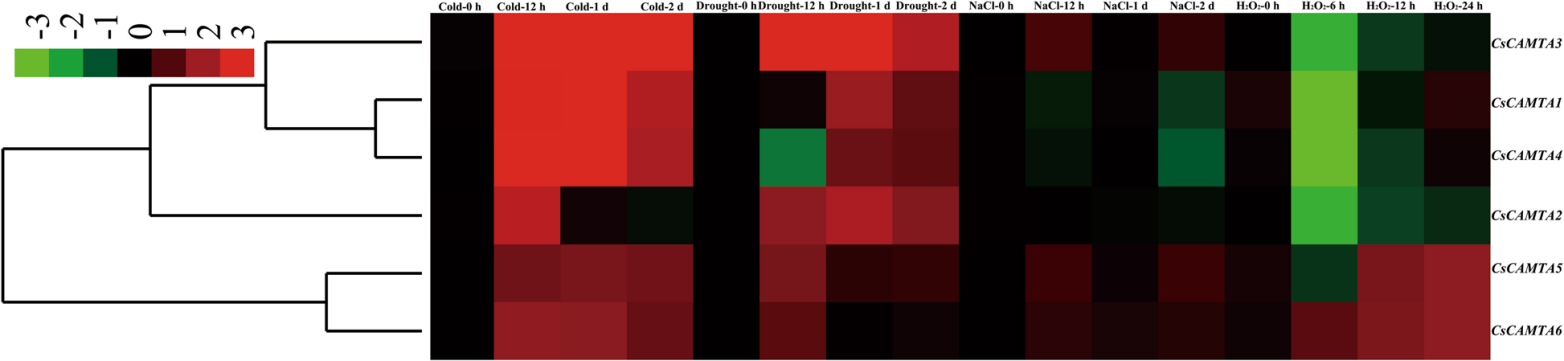
Expression patterns of *CsCAMTAs* under various abiotic stresses conditions. Samples at 0 h were set as control, and the data were calculated by using 2^–ΔΔCt^ method. The red and green colors represent higher and lower expression levels, respectively. The colorbar was presented on the upper-left of the heat map

To elucidate the spatial–temporal expression patterns of *CsCAMTAs* under various abiotic stress conditions, the transcription abundances of *CsCAMTAs* were detected. As shown in Fig. 6, *CsCAMTAs* were differentially expressed under different stress conditions. Specifically, all of *CsCAMTAs* were remarkably induced by CT, and their expression levels were more than twofold higher than those at 0 h of CT. In particular, the expression level of *CsCAMTA4* was more than 30-fold higher after 12 h of CT, and the expression levels of *CsCAMTA1*/*2* were also more than 30-fold higher after 1 d of CT. Similarly, the expressions of all *CsCAMTAs* were also up-regulated by DT at different treatment time points. Among them, *CsCAMTA2* was significantly up-regulated by DT, which showed more than fourfold high expressions throughout entire DT period, especially more than 14-fold higher after 12 h of DT than those at 0 h of DT. Meanwhile, the expression level of *CsCAMTA3* was induced more than twofold higher after 2 d of DT treatment. In contrast, the expressions of all *CsCAMTAs* were slightly influenced by NT, of which the expressions of *CsCAMTA1*/*3*/*4* were reduced, but the other genes were induced by NT in some degrees at different processing time points. A similar result was also obtained by H_2_O_2_ treatment, where we found *CsCAMTA1*-*4* transcripts were decreased, while *CsCAMTA5* and *CsCAMTA6* transcripts were increased within 1 d of H_2_O_2_ treatment, except for 6 h.

### Expression analysis of *CsCAMTAs* under hormone treatment conditions

**Fig. 7:**
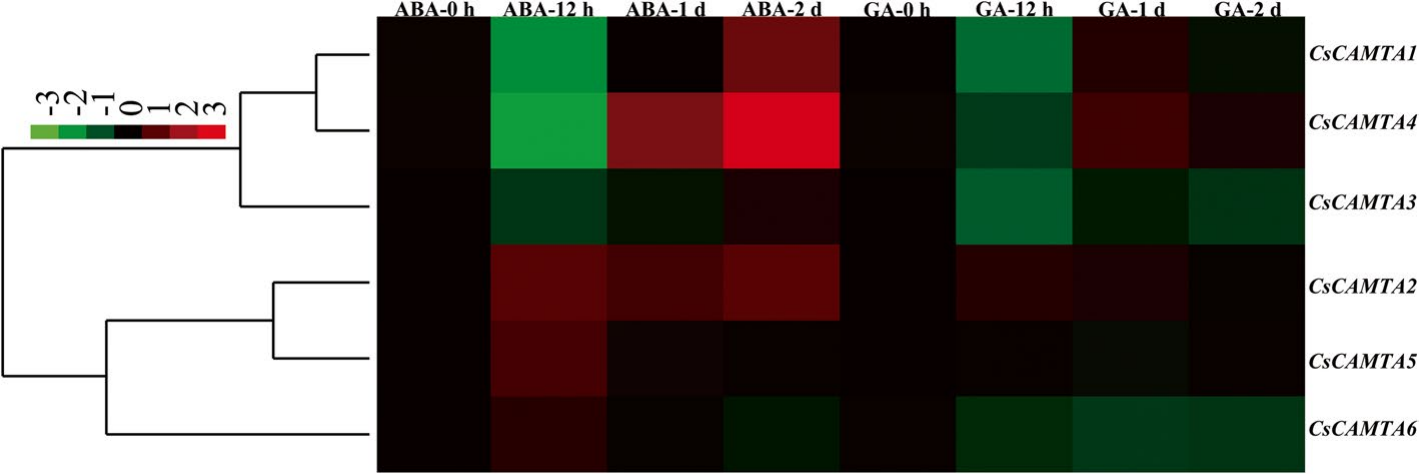
Expression patterns of *CsCAMTAs* under hormone treatments conditions. Samples at 0 h were set as control, and the final results were calculated by using 2^–ΔΔCt^ method. The red and green colors represent higher and lower expression levels, respectively. The colorbar was presented on the upper-left of the heat map

To explore the roles of *CsCAMTAs* in responding to hormone treatments, their expressions were analyzed under ABA and GA treatment conditions. Under ABA treatment condition, we found that the expressions of *CsCAMTA1*/*4* were down-regulated firstly within 12 h of ABA treatment, and then up-regulated with the ABA treatment prolonged. In particular, *CsCAMTA1*/*4* transcripts respectively showed more than 2- and sixfold higher after 2 d of ABA treatment than those at 0 h of ABA treatment (Fig. 7). In contrary, *CsCAMTA5/6* were up-regulated firstly within 12 h of ABA treatment, and then down-regulated with the ABA treatment prolonged. In addition, *CsCAMTA2* showed nearly twofold higher expressions within 2 d of ABA treatment, while *CsCAMTA3* transcripts seemed to be not affected by ABA treatment. In contrary to ABA treatment, the expressions of *CsCAMTAs* were slightly influenced by GA treatment. Among them, *CsCAMTA1*/*4* transcripts decreased within 12 h of GA treatment, but increased after 1 d of GA treatment, while *CsCAMTA3*/*6* transcripts decreased within 2 d of GA treatment. Besides, the expressions of *CsCAMTA2* were slightly induced within 1 d of GA treatment, and then deduced until to 2 d of GA treatment. However, *CsCAMTA5* was not affected by GA treatment (Fig. 7).

### Expressions analysis of *CsCAMTAs* during CA periods

**Fig. 8:**
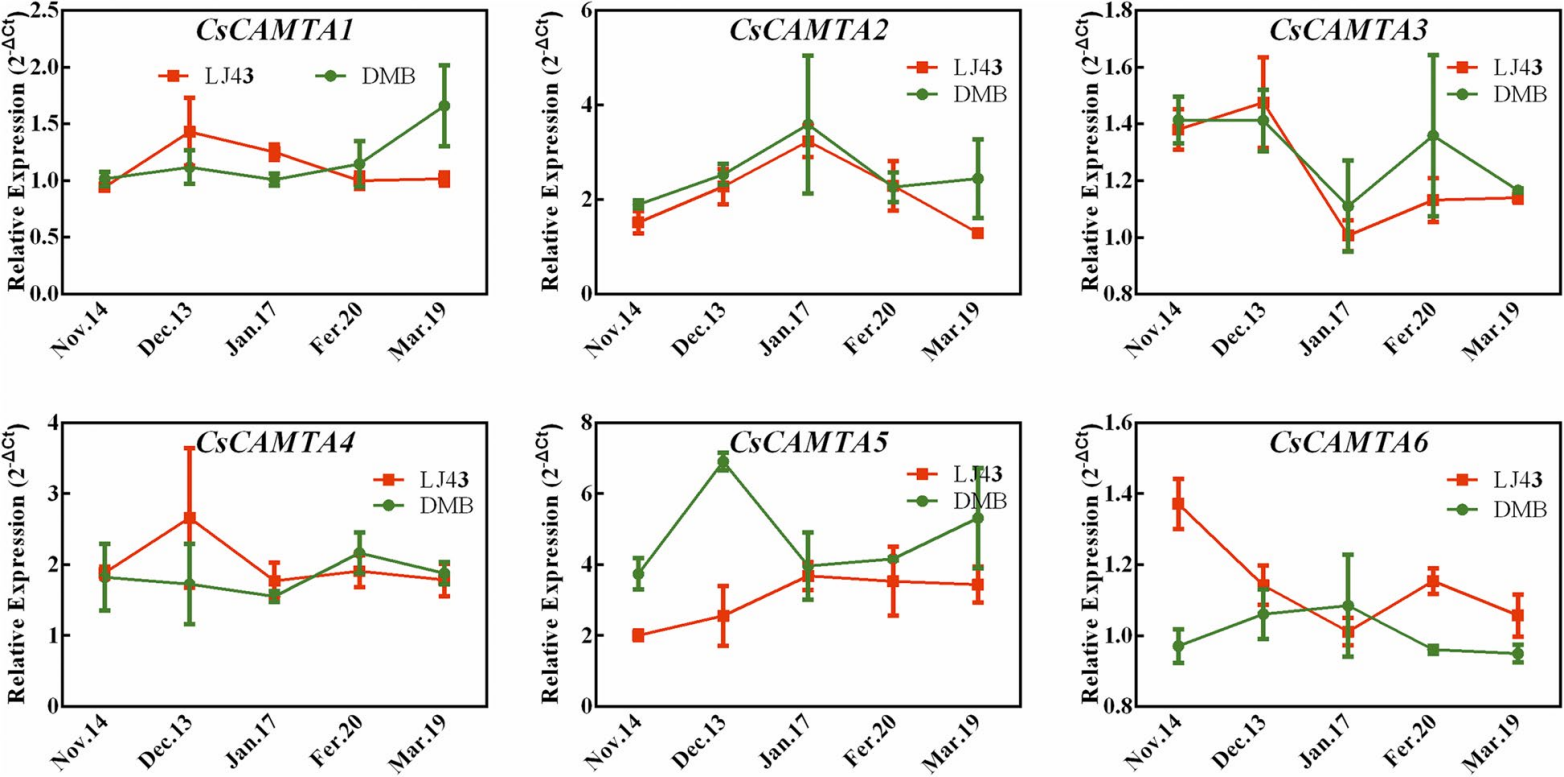
Expression profiles of *CsCAMTAs* in the mature leaves of two-tea cultivars during CA periods. ‘DMB’ and ‘LJ43’ represent ‘DaMianBai’ and ‘LongJing43’ tea plant cultivars, respectively. The relative expression levels were calculated with 2^–ΔCt^ method. Data are shown as the means ± SE (n = 3)

As above mentioned, due to *CsCAMTAs* transcripts were remarkably induced by cold treatment, we further explored their expressions patterns between the cold-resistant cultivar ‘LongJing43’ and the cold-susceptible cultivar ‘DaMianBai’ under CA condition (Fig.S2). As Fig. 8 shown, the expression patterns of *CsCAMTAs* were varied from each other and also varied in these two tea plant cultivars during CA periods. In terms of ‘LongJing43’, with the exception of *CsCAMTA6*, the other genes were up-regulated with the temperature decreased from November 14^th^ to December 13^th^ (CA stage), and then the expressions recovered to normal levels with the temperature raised from February 20^th^ to March 19^th^ (de-CA stage). Similarly, *CsCAMTA1*/*2*/*5*/*6* transcripts increased at CA stage, while recovered to normal levels at de-CA stage in ‘DaMianBai’. In addition, the expression levels of *CsCAMTA1*/*3*/*4*/*6* were higher in ‘LongJing43’ than in ‘DaMianBai’ at CA stage, while *CsCAMTA2*/*5* showed higher expression levels in ‘DaMianBai’ than in ‘LongJing43’ during entire CA periods, suggesting that the differential expression patterns of *CsCAMTAs* may be positively contributed to the cold resistance of tea plant. However, the correlation between the expressions of *CsCAMTAs* and the cold resistance of tea plant needs to be further explored in future.

## Discussion

### CsCAMTAs possess similar biological characteristics with other CAMTAs of various plant species

As a type of signal responsive proteins or ethylene-induced CaM-binding proteins, CAMTAs are known as the largest and best characterized CaM-binding TFs [3]. Currently, numerous of CAMTAs have been identified from more than 20 kinds of plant species, such as maize [18], bananas [21], wheat [25], flax [27], *Arabidopsis* [59], etc. Further research found that CAMTAs were constituted with 6 highly conserved functional domains across the species, including nuclear localization signals (NLS), CG-1 domain, TIG domain, CaMBD, ANK repeat and IQ motif [12]. As well known that CG-1 domain was contributed to binding DNA directly and activating transcription, TIG was associated with the interaction to TFs through nonspecific DNA binding, ANK was function to protein–protein interaction, and IQ motif was correlated to the binding of CaM and CaM-like proteins [12]. In this study, basing on the HMM models of CG-1 domain, IPT/TIG domain, Ankyrin repeat, and IQ domain, total of 6 CsCAMTAs were identified from tea plant genome, and all of them shared closet relationship with NtCAMTAs. Besides, each CsCAMTA possesses the above mentioned functional domains. In particular, the NLS was detected in CG-1 domains of all CsCAMTAs, which further confirmed the nucleus localization of CsCAMTAs. However, the numbers of ANK repeats and IQ motifs were varied among these CsCAMTAs, suggesting that CsCAMTAs might interact with different numbers of proteins or form different numbers of heteromeric (or homomeric) complexes through their ANK domains, meanwhile, CsCAMTAs might bind to different numbers of CaM or CaM-like proteins. Similarly, many CAMTAs in other species, such as FaCAMTA [60], DzCAMTAs [61], LuCAMTAs [27], and MuCAMTAs [21], etc. had also been demonstrated to perform similar functions. In terms of the gene structures of *CAMTAs*, many studies have found a fixed number of introns and exons existed in *CAMTAs* gene family members. In this study, the introns numbers of *CsCAMTAs* were varied from 10 to 12, which was similar to the genes structures of *CAMTAs* in *Arabidopsis* [59], maize [18] and tomato [20], respectively. This result was also similar to the result of phylogenetic tree, suggesting that *CAMTAs* are relatively conserved among different species in the permanent evolution.

Gene duplication events, including tandem replication and segment duplication, are the main pathways that involved in the expansion of gene family members. Tandem replication events have also been demonstrated to contribute to improving the stress-resistance of plants in dealing with various environment stresses [62]. In our study, the tandem replication events were not found in *CsCAMTAs* gene family, but two segment duplication events were identified, which suggested that segment duplication events might be the major pattern for the expansion of *CsCAMTAs* genes family in tea plant. The similar results have also found in banana [21], *Durio zibethinus* [48], and *Cucurbita moschata* and *Cucurbita maxima* [51], where they found that almost all of the identified *CAMTAs* were located on different chromosomes in different species. At present, there is no study on the collinearity analysis of *CAMTAs* between different species. Based on the results of the synteny analysis in our study, we found different numbers of orthologous gene pairs of *CsCAMTAs* were identified between different species. Similar to our results, many gene families, such as *CsNACs* [63], *CsMYBs* [64], *CsACSs* [65], and *FtHsfs* [66] and so on, have also been found possess different numbers of orthologous gene pairs between different species though synteny analysis, which suggested the divergence evolutionary existed in different species.

### *CsCAMTAs* mediate vegetative and reproductive progress of tea plant

Numerous of studies have showed that *CAMTAs* are widely involved in the plant vegetative and reproductive processes, especially in leaf senescence, flowering and fruit development. As demonstrated by Yang et al. (2012) [20], 7 *SlSR*/*CAMTAs* mediated fruit development and ripening of tomato, and *SlSRs* differently expressed in different tomato tissues, different fruit development stages and in a tomato ripening mutant (*rin*). Most notably, the transcription abundances of *SlSR2* were too low to detect at the mature green and breaker stages, while *SlSR3L* and *SlSR4* expressed highly in fruit tissues. Besides, *SlSRs* were rapidly induced by ethylene treatment in mature green stage fruit, of which the expressions of *SlSR1* increased about fourfold higher after 2 h of ethylene treatment, which indicated that *SlSRs* served as early ethylene responsive genes mediate fruit ripening through ethylene-dependent pathway [20]. A similar result was also obtained by Yang et al. (2000) [67], where they found a *CAMTA* gene, *NtER1*, transcripts were higher in fully opened flowers, senescing flowers, senescent leaves than that in immature, fully mature leaves and buds. In addition, *NtER1* transcripts were rapidly induced after 15 min of exposure to ethylene in tobacco flowers at different development stages, suggesting *NtER1* was an early ethylene-up-regulated gene [67]. In *Cucurbita maxima* and *Cucurbita moschata*, all *CmoCAMTAs* and *CmaCAMTAs* showed higher expression levels in roots than that in stem, leaf, and fruit tissues, meanwhile, the expression levels of *CmaCAMTA1*-*6* were higher in fruit than that in leaf, implicating that *CmaCAMTAs* mediated fruit development [68]. As CAMTAs served as the downstream targets of CaM, in order to confirm whether calcium/calmodulin signaling participated in fruit ripening, Yang et al. (2015) further analyzed the expressions of *SlCaMs* during fruit development and ripening, and thus they found all *SlCaMs* had a peak expression pattern at 10–30 days after anthesis and at turning/pink stages, respectively [22]. Besides, *SlCaMs*, especially *SlCaM2*, were also stimulated by ethylene treatment. In addition, *SlCaM2* overexpressed transiently in mature green fruit could delay ripening, while retarding *SlCaM2* expression would promote ripening, which indicated that *SlCaM2* could be a major regulator involved in the modulation of fruit ripening [69]. These results further confirmed the functions of Ca^2+^-CaM-CAMTAs complexes in dealing with plant vegetative and reproductive processes. At present, there have 5 Calmodulin-like (CML) proteins (CsCMLs), been isolated and functionally characterized in tea plant. Expression analysis results showed that *CsCML16*/*18*–*1* presented remarkable expression levels in flowers than in other tissues, suggesting that *CsCMLs* possess tissue-specific expression in tea plant [70]. Correspondingly, we found that 6 *CsCAMTAs* were expressed differentially in various tea plant tissues, of which the highest expression levels were detected in senescent leaves, flower and root than that in other tissues, indicating that *CsCAMTAs* were developmentally regulated and acted as triggers for senescence and death. However, whether the similar expression patterns exist in *CsCaMs* family genes in different tea plant organisms still needs to be further studied in future, as the gene numbers, tissue-specific and spatial–temporal expression patterns of *CsCaMs* have not been explored until now. Besides, as a type of EICBPs, whether CsCAMTAs served as ethylene responsive genes to mediate leaf senescence also needs to be further studied.

### *CsCAMTAs* involved in various abiotic stresses and hormones responses

Apart from mediating plant developmental biology, *CAMTAs* also play critical roles in regulating biotic and abiotic stresses responses, such as diseases, pests, drought, salt, low temperature etc. [29, 59]. For example, 6 *AtCAMTAs* have been reported to be quickly and differentially stimulated by various stresses and hormones [11, 29, 71, 72]. Similarly, 9 *ZmCAMTAs*, 9 *CiCAMTAs*, and 15 *TaCAMTAs* were respectively stimulated by various hormones, abiotic and biotic stresses [18, 24, 25]. It is now known that CAMTAs-regulated genes depend on Ca^2+^ signals, the Ca^2+^-CaM-CAMTAs complexes could nonlinearly amplify different calcium signatures, and then the calcium signatures are decoded to produce specific CAMTA-regulated gene expression responses [73]. In recent years, the stress responses regulation mechanisms of CAMTAs have been partially elucidated in *Arabidopsis* based on the overexpression and mutation techniques. Under stress stimuli condition, CAMTAs regulate the gene expressions of downstream targets through specifically binding to the core motifs (A/C) CGCG (C/G/T) or (A/C) CGTGT contained in the promoter regions of lots stress-responsive genes. It is well known that the ICE-CBF-COR signaling pathway plays the leading role in enhancing cold tolerance of plants upon exposure to nonfreezing temperatures. In *Arabidopsis*, 3 *AtCBFs* could be induced within 15 min, and reached the highest level after 3 h of cold treatment when exposed to low temperature. Besides, *AtCBF1*-*3* genes mediate 414 *COR* genes expressions, including 346 CBF-activated genes and 68 CBF-repressed genes when exposed to nonfreezing temperature, indicating that *CBF* genes play central role in CA [74]. Apart from CBF regulons, CAMTAs have been identified as transcription activators of CBFs in *Arabidopsis*, which specifically binding to conserved DNA motif 2 (CM2, vCGCGb) in the promoter of *CBF2*. Among them, CAMTA3 referred as a positive regulator, mutation of *AtCAMTA3* resulted in approximately 50% reduction of *CBF2* expression, and a much higher extent were observed in double *camta1 camta3* mutant plants, which indicated that CAMTA proteins play positive roles in cold acclimation [59]. Further research found that AtCAMTA1 and AtCAMTA2 cooperated with AtCAMTA3 induced the expressions of *CBF1*, *CBF2* and *CBF3*, following upregulated the expression of approximately 15% genes that independent CBF pathway after exposed to 4 °C for 24 h, and thus resulted in enhancing plant freezing tolerance [31]. Moreover, Kidokoro et al. (2017) found that AtCAMTA3/5 could induce the expression of *DREB1B* under rapid temperature reduction condition [75]. Meanwhile, AtCAMTA3/5 sustained this effect throughout the day and night, in contrast to CIRCADIAN CLOCK ASSOCIATED1 (CCA1) and LATE ELONGATED HYPOCOTYL (LHY), which only promoted the upregulation of *DREB1B* only during the day [75]. In terms of tea plant, it has also revealed that calcium signaling pathway plays critical role in improving cold tolerance during CA condition [26]. In our research, we found the expression levels of all *CsCAMTAs* were significantly induced by cold treatment. Meanwhile, *CsCAMTAs* showed differential expressions between cold-resistant and cold-susceptible tea plant cultivars during CA periods. At the same time, the expressions of *CsCBFs* genes in tea plant have also been confirmed to up-regulated by low temperature. Recently, 6 *CsCBFs* genes have been isolated from the chromosome-level genome of tea plant [76]. As Hu et al. (2020) demonstrated that the yeast cells containing *5 pGBKT7-CsCBFs* (*pGBKT7-CsCBF2*-*6*) recombinant plasmids grew well on the selection media and positive for α-galactosidase activity respectively, which indicated that CsCBF2-6 possess transcriptional activity [76].

Expression analysis results found that the transcriptions of *CsCBFs* were differentially regulated by various abiotic stresses and hormone treatments. Notably, these *CsCBFs* genes were markedly up-regulated, and the expression levels of *CsCBF1*-*3* were up-regulated more than 100-fold or even 1000-fold within 1 d of cold treatment. In addition, overexpression of *CsCBF3* could enhance the cold tolerance of transgenic *Arabidopsis* potentially through an ABA-independent pathway [76]. In the present study, we also searched the putative core CsCAMTAs binding motifs, (A/C) CGCG (C/G/T) or (A/C) CGTGT, in the promoters of *CsCBF1*-*6*. As shown in Table S7, each of *CsCBFs* contains different numbers of (A/C) CGCG (C/G/T) and (A/C) CGTGT motifs, which indicated that CsCAMTAs are served as a type of transcription activators of *CsCBFs*, which could specifically bind to the conserved (A/C) CGCG (C/G/T) or (A/C) CGTGT motifs in the promoters of *CsCBFs* to regulate their expressions, and thus mediate the cold response of tea plant.

In plants, CAMTAs also involved in response to drought and salinity. As Pandey et al. (2013) demonstrated that AtCAMTA1 mediated drought responses in *Arabidopsis* [29]. Microarray analysis found that the expressions of many genes involved in DNA methylation, stress response, apoptosis, photosynthesis and osmotic balance were greatly altered in *camta1* mutants under drought conditions. Specifically, several stress responsive genes, including *RD26*, *Early-responsive to dehydration 7* (*ERD7)*, *Ras-related protein* (*RAB18)*, *Lipid protein* (*LTPs)*, *Clod related protein* (*COR78*), *CBF1*, *Heat shock proteins* (*HSPs*) etc., were positively regulated by AtCAMTA1, and the conserved (A/C) CGCG (C/G/T) or (A/C) CGTGT motifs enriched in the promoters regions of these genes, suggesting that AtCAMTA1 mediated drought recovery mainly through regulating the expressions of AP2-EREBP transcription factors and depending on ABA signaling pathway [29]. In addition, overexpression of *GmCAMTA12* in *Arabidopsis* and soybean respectively enhanced drought tolerance of transgenic lines. Under drought stress condition, the expression of *AtAnnexin5*, *calmodulin binding heat shock protein* (*AtCaMHSP*), *At2G433110* and *AtWRKY14* were up-regulated in transgenic *Arabidopsis*. Similarly, the expressions of *elongator complex* (*GmELO)*, *nucleic acids binding* (*GmNAB*) and *phospholipase A1-IId* (*GmPLA1*-*IId)* were significantly up-regulated in transgenic soybean hairy roots when exposed to drought stress condition, and the conserved (A/C) CGCG (C/G/T) or (A/C) CGTGT motifs were also enriched in the promoter regions of these genes [77]. For tea plant, it has been known that the application of exogenous ABA could promote drought resistance of tea plant, suggesting that drought response of tea plant relies on ABA signaling pathway [78, 79]. Under drought stress condition, 12 TF families members (*bZIP*, *NAC*, *squamosa promoter-binding protein-like* (*SPL*), *APETALA2/Ethylene-responsive element binding proteins* (*AP2*/*EREBP*), *Basic helix loop helix* (*bHLH*), etc.) and numbers of genes involved in ABA biosynthesis and signaling (*9-cis-epoxycarotenoid dioxygenase 1* (*NCED1*), *NCED4*, *pyrabactin resistance 1-like 4* (*PYL4*), *PYL8*, *PP2C1*-*6*, *sucrose non-fermenting1-related protein kinase 2.2* (*SnRK2.2)*, *SnRK2*.*3*, *SnRK2*.*5*, *SnRK2*.*6*, etc.), carbohydrate metabolism (UDP-glucose pyrophosphorylase (*UDPGase*), sucrose-phosphate synthase (*SPS*), trehalose phosphate synthase (*TPS*), trehalose phosphatases (*TPP*), mannose-6-phosphate reductase (*M6PR*), and mannose-1 phosphate phosphatase (*M1PP*) were up-regulated [80]. In this study, we found all *CsCAMTAs* were up-regulated by DT at different treatment time points, which indicated that *CsCAMTAs* collaborates with other TFs family members to participate in regulating drought response of tea plant. However, the regulation mechanism of *CsCAMTAs* needs to be further explored.

Recently, Shkolnik et al. (2015) found that *AtCAMTA6* contributed to controlling Na^+^ homeostasis in germinating seedlings of *Arabidopsis* through ABA-dependent and-independent signaling pathways [72]. However, another *CAMTA* gene, *AtCAMTA3*, was reported as a negative regulator of salt tolerance by directly repressing salt-responsive genes transcripts, mutation of *AtCAMTA3* resulted in higher salt tolerance in *camta3* mutants than the wide type and complemented line [81]. Yuan et al. (2021) found that all *CmoCAMTAs* in the leaf vein were remarkably induced, while all *CmoCAMTAs* in leaf mesophyll were inhibited by salt stress [68]. In terms of tea plant, transcriptomic analysis revealed that many TFs genes (*e.g. bZIP*, *HD*-*Zip*, *APETALA2/ethylene-responsive factor* (*AP2*/*ERF)*, *WRKY*, *NAC*, *MYB*, *bHLH* and *zinc finger*-*TFs*) and many genes involved in Ca^2+^ signal transduction (*e.g. CaM4*, *CDPK7/3/15/16,* and *CML18/20/49*), ABA signaling pathway (*e.g. type 2C protein phosphatase* (*PP2C*) *2/3/12/27/14/51/54/60*), and mitogen-activated protein kinase (MAPK) cascades pathway (MAPK *kinase* (*MAPKK*) 2/4/5) were differentially expressed in tea plant under slat stress condition. In Ca^2+^ signal transduction pathway, 3 *CaMs/CMLs* genes (*CML20*, *CML18*, and *CML49*) were up-regulated, whereas *CaM4* was down-regulated by salt stress [82]. At present study, partial of *CsCAMTAs* were slightly induced by NT, which indicated that Ca^2+^-CaM-CAMTAs complexes also play a role in salt response of tea plant. However, the specific downstream targets of CsCAMTAs need to be further explored.

At present, it has been clear that the expressions of many *CAMTAs* in different plant species could be stimulated by various hormones, such as auxin, ABA, ethylene, methyl jasmonate (MeJA), and SA, etc. [18, 24, 25, 31]. For example, *CAMTA1-3* repressed the expressions of *isochorismate synthase 1* (*ICS1)*, *CBP60g* and *SAR deficient 1* (*SARD1*), and thus inhibited SA biosynthesis in *Arabidopsis* under warm temperature condition [31]. In the present study, we found the expressions of 5 *CsCAMTAs* (*CsCAMTA1/2/4/5/6*) were induced or reduced by exogenous ABA at different treatment time points. Meanwhile, GA treatment also slightly affected the expressions of 5 *CsCAMTAs* (*CsCAMTA1-4/6*) within 2 d of treatment. Correspondingly to these results, many hormone-related *cis*-acting elements were enriched in the promoter regions of *CsCAMTAs*, suggesting that the expressions of *CsCAMTAs* were regulated by hormone signaling pathways.

## Conclusion

Total of 6 *CsCAMTAs* genes were identified from tea plant genome of ‘ShuChaZao’ cultivar. Each CsCAMTA was predicted to contain 6 conserved functional domains and located in nucleus. All CsCAMTAs showed closest relationship with NtCAMTAs except for CsCAMTA4. *CsCAMTAs* may be mediated tea plant vegetative and reproductive processes, especially in aging and flowering periods. Besides, *CsCAMTAs* were widely involved in various abiotic stresses and hormones responses, among of which *CsCAMTAs* may be contributed to improve cold tolerance of tea plant depending on CBF signaling pathway. In addition, *CsCAMTAs* were differentially expressed between cold-resistant cultivar ‘LongJing43’ and cold-susceptible cultivar ‘DaMianBai’ during cold acclimation periods, suggesting that *CsCAMTAs* may be served as potential molecular markers for screening tea plant germplasms with cold resistance. Overall, the present study provided theoretical support for deeply exploring the regulation mechanisms of *CsCAMTAs* in tea plants.

## Supplementary Information

**Additional file 1:** **Fig. S1.** The chromosomal distributions of *CsCAMTAs* in ‘HuangDan’ and ‘TieGuanYin’ cultivars. A: The chromosomal distribution of CsCAMTAs in ‘HuangDan’ genome. B: The chromosomal distribution of CsCAMTAs in ‘TieGuanYin’ genome.

**Additional file 2:** **Fig. S2.** The diurnal dynamic changes of temperature from November 2018 to March 2019 in Hangzhou.

**Additional file 3:** **Table S1.** All sequences used to construct phylogenetic tree.

**Additional file 4:** **Table S2.** Primers information used in qRT-PCR detection.

**Additional file 5:Table S3.** The chromosomal distribution positions in chromosomes of three tea plant cultivars.

**Additional file 6:** **Table S4.** The synteny analysis results of *CsCAMTAs* within ‘ShuChaZao’ genome.

**Additional file 7: Table S5.** The Ka-Ks ratios of *CsCAMTAs.*

**Additional file 8:** **Table S6.** The synteny analysis results of *CsCAMTAs* between different species.

**Additional file 9:** **Table S7.** 2000-bp promoter sequences of *CsCBFs*.

## Data Availability

The datasets generated and/or analyzed during the current study are available in this article and the additional files. The nucleotide and protein sequences of CAMTA-related genes in *Arabidopsis*, *Oryza sativa*, *Populus trichocarpa*, *Malus pumila*, *Triticum aestivum*, *Nicotiana tabacum* and *Zea mays* are available in Phytozome v13 database (JGI, https://phytozome.jgi.doe.gov/pz/portal.html).
