# Supplementary material for: Genome-wide identification and expression analysis of the calmodulin-binding transcription activator (CAMTA) family genes in tea plant
Source: BMC Genomics. 2022 Sep 22;23:667. doi: 10.1186/s12864-022-08894-x (PMC9502961; doi:10.1186/s12864-022-08894-x)
Supplement: Supplementary file 1 — Additional file 1: Fig. S1. The chromosomal distributions of CsCAMTAs in ‘HuangDan’ and ‘TieGuanYin’ cultivars. A: The chromosomal distribution of CsCAMTAs in ‘HuangDan’ genome. B: The chromosomal distribution of CsCAMTAs in ‘TieGuanYin’ genome. [file 12864_2022_8894_MOESM1_ESM.docx]

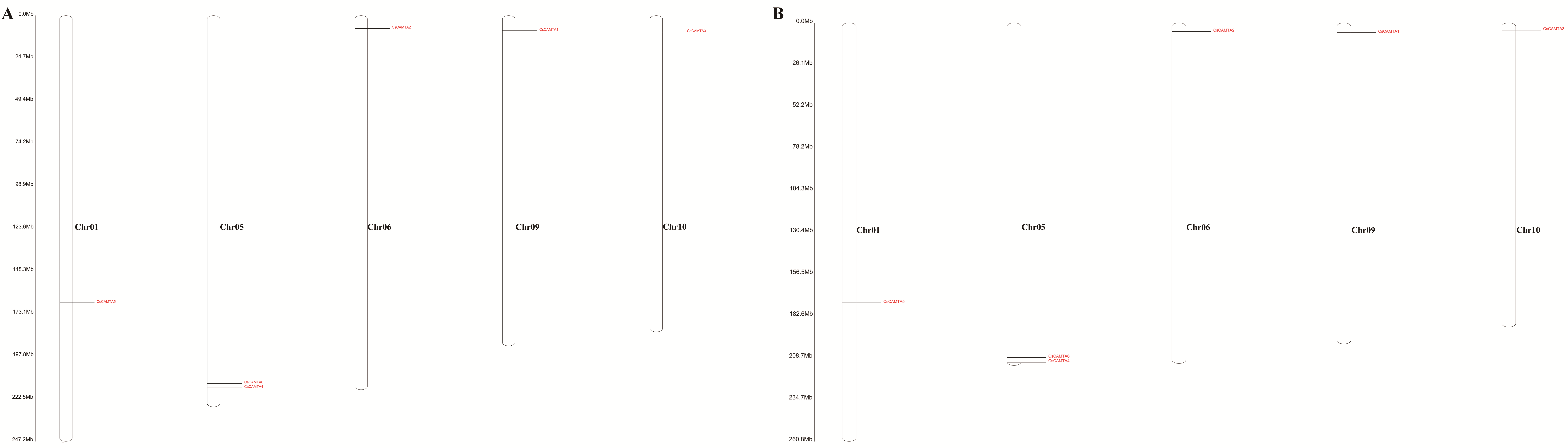


Fig. S1. The chromosomal distributions of *CsCAMTAs* in ‘HuangDan’ and ‘TieGuanYin’ cultivars.

A: The chromosomal distribution of CsCAMTAs in ‘HuangDan’ genome. B: The chromosomal distribution of CsCAMTAs in ‘TieGuanYin’ genome.
