# Supplementary figures and images for: Genome-wide identification and expression analysis of the calmodulin-binding transcription activator (CAMTA) family genes in tea plant
Source: BMC Genomics. 2022 Sep 22;23:667. doi: 10.1186/s12864-022-08894-x (PMC9502961; doi:10.1186/s12864-022-08894-x)

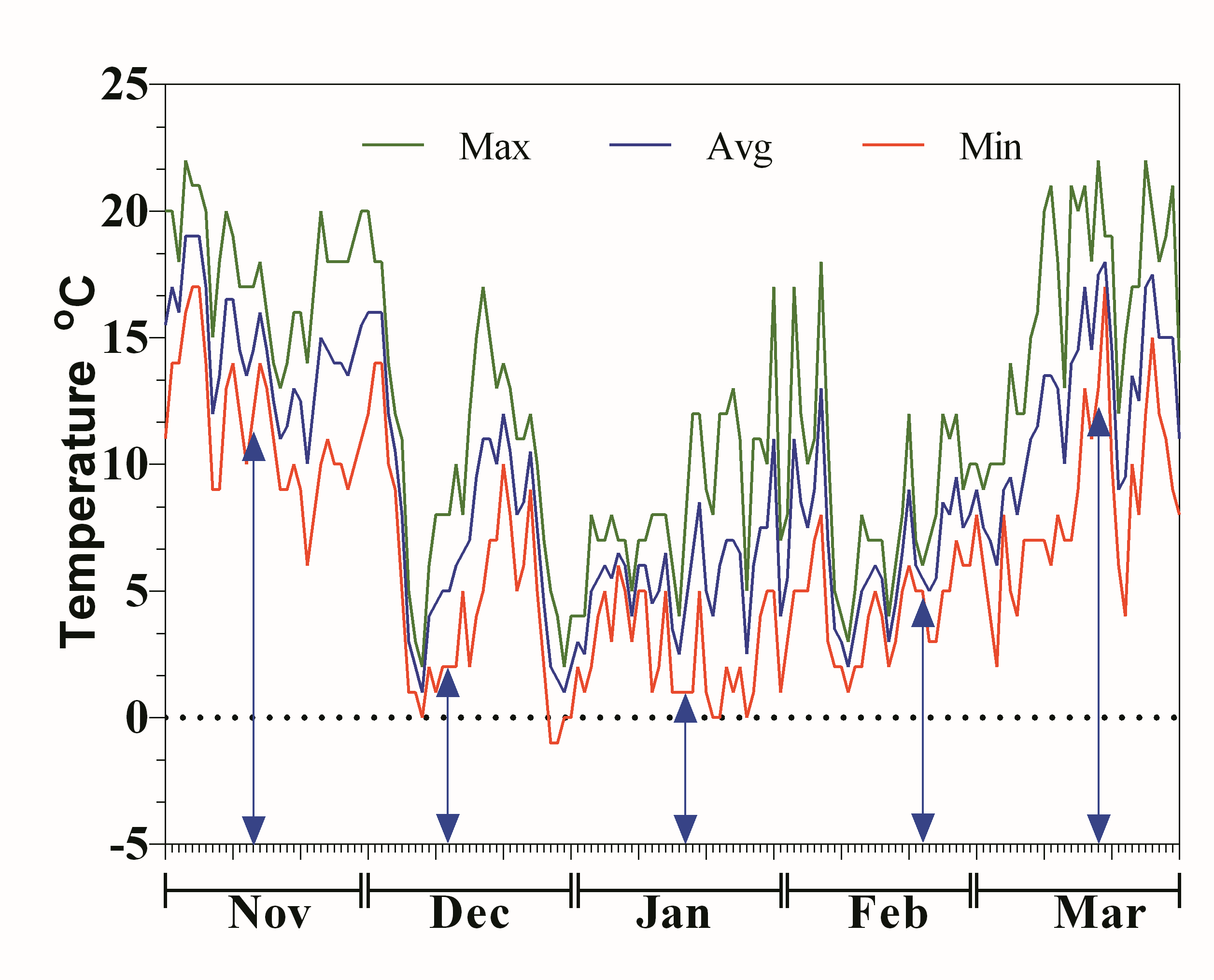


Fig. S2. The diurnal dynamic changes of temperature from November 2018 to March 2019 in Hangzhou.

Supplement: Supplementary file 2 — Additional file 2: Fig. S2. The diurnal dynamic changes of temperature from November 2018 to March 2019 in Hangzhou. [file 12864_2022_8894_MOESM2_ESM.docx]
