# Supplementary material for: Genome-wide identification and expression analysis of the calmodulin-binding transcription activator (CAMTA) family genes in tea plant
Source: BMC Genomics. 2022 Sep 22;23:667. doi: 10.1186/s12864-022-08894-x (PMC9502961; doi:10.1186/s12864-022-08894-x)
Supplement: Supplementary file 4 — Additional file 4: Table S2. Primers information used in qRT-PCR detection. [file 12864_2022_8894_MOESM4_ESM.docx]

**Table S2** Primer information used in qRT-PCR detection.

| **ID** | **Gene name** | **Forward/Reverse** | **Primer sequence (5' to 3')** |
| --- | --- | --- | --- |
| XP_028075461.1 | *CsCAMTA1* | Forward | AGCTGCAGAAAACGAGAAGCTCTCTG |
|  |  | Reverse | GGGCTACTCTCTTCATCACCTCTTCC |
| XP_028093707.1 | *CsCAMTA2* | Forward | CCCGTTAGGTGGTTATGGTGTTCC |
|  |  | Reverse | TTGGTTTGGTTGCTGCATAGACTG |
| XP_028051249.1 | *CsCAMTA3* | Forward | CATGGGTGGTCTGTATCAAGAAGAT |
|  |  | Reverse | CATGCGGCGAAGCAGCCAAGGTT |
| XP_028068006.1 | *CsCAMTA4* | Forward | TCGACAAGGCTCTTTGACAC |
|  |  | Reverse | ATTACCGCTACCACCACCTC |
| XP_028069892.1 | *CsCAMTA5* | Forward | GCTGTTCGTGGTGTTGAGATGC |
|  |  | Reverse | AGGCTCTGAGGTGAGGAAGTCT |
| XP_028094568.1 | *CsCAMTA6* | Forward | CTGCTCCTGATACCGCATGAAAATTAC |
|  |  | Reverse | TGAGTGCAAGTCCTTGGAGGTTCACA |
| GAAC01052498.1 | *CsPTB* | Forward | TGACCAAGCACACTCCACACTATCG |
|  |  | Reverse | TGCCCCCTTATCATCATCCACAA |
