# Supplementary material for: Genome-wide identification and expression analysis of the calmodulin-binding transcription activator (CAMTA) family genes in tea plant
Source: BMC Genomics. 2022 Sep 22;23:667. doi: 10.1186/s12864-022-08894-x (PMC9502961; doi:10.1186/s12864-022-08894-x)
Supplement: Supplementary file 9 — Additional file 9: Table S7. 2000-bp promoter sequences of CsCBFs. [file 12864_2022_8894_MOESM9_ESM.docx]

**Table S7. 2000-bp promoter sequences of CsCBFs.** The putative (A/C) CGCG (C/G/T) motif was highlighted with yellow color and the (A/C) CGTGT motif was highlighted with red color.

>CsCBF1

TACAACTATCAAATCAATTAAAAAAAAAAGTTAAAATTTATCTCAAACAT

AATTGTTTTAACCAAGATTTAAAAAGTCAAAAAGTCATACTAAATTATAC

CAAACGAGGGTTTAAAAAGCTCGGGATCAACCTCATCGAGGCTATTTGAA

TCAGCTATTTTTTTTTTTTTTTATATTTCGAGTGATAGCATAAATCTTGA

AAATCAATCGTAGGGCATTAATTGTCTGAACATAATCGGAAAATAAGAAG

ACCCGTATTAGATTACAACATTCATTACCAACTAGACAATGCGAAACTTC

ACATAAGAAATTCAACTACATTTTAGATACACACAAAACATTCATCTATA

TTATTATATTAAGGTCTCGTTTGAGAATAGGTTAATTTAGTAATATTTTG

ATTATTTGGCTAAAATATAAGCTAAAAAAAACTAGTATGTTTGGGATGAC

TTTTTGACTTTTTATGAAAAAATGTGCAAAAAGTAGAGAGAAAAAAGAAT

CTTTGACTTTTTGATATGAGTTGTTGGCTTTTTGACTTTTTTAGTAACTT

TTTGATTTTTTAACTAAACCGCCCTGTTGCCAAACGAGGCCTAAGTATAG

ACTAAACTCGGTGTTTAAACATTTGTTTGTTTCTCCCTACGCCACATGTT

CCTCAGTTTTCTTATTTCAAAATAAAATATATAAATAAGAGAAGTGAGAC

TTGAATCTTAGATTTTTCAAAGAAGACTCTTACATACAACATATAGTAAA

TATTTTATTTTATTTTATTTGAATTGTATACACTTTTCGTATACCAACCA

CTAATATGAGTAAGAGAGAATATATATACAGGTGAGAGTTAGGAAAAAAT

TATAAAATATATGGGAGGTGCAAGTGTGTTAGCAGTGAGATGCAAATGAA

AGATTGTAAAAATACAACCCCTCTGTATATTAGTAATTTTCCCCCCTAAT

TTCTATCTACGGGTGGTTTCCCTTATGAATAATGAGAAAGAATAAAGATT

GGGACCCCTTTTCCTCCCACCCCCTTTGACTCTCTTTCTTTTTTGATTGA

TCACTTTTTTTTTTTTTTTTTTTCTTCTCCTTCAATCACCACCCACCACA

TATGTATAAAGAGGTGGTGAGGATTGAAATTTGAAGGAATCTAAATCACT

ACCGAATGAGAAATACAGTTTAAGATGTGAAAATTCATATTGAAACACCC

ACTATTATTTGATAGGGCACCTTTGGTATAATTGTGAGGAATTTGTCTAT

TGAAAACTGTAACAAGGACAGAATCCCCAACGGGGCAATGTTTGACTCAG

ACGTCAGCAATCTTCCTATCCACGGAATTCAGGAGATTCCACACCATACA

ATGAATTTTCAACTAATAAATAATAAGTTGGAGTATCTTATGAATGTCCT

TATATATTTTAAATAATTATTAAATATTTTATATAATATTCATATAAATG

TAAGATTTATGTATTTATTATATAAAATATTTAATAATTTATTTGACATT

TTTGGTTTACAATTTTTTTGGTGTGACAGGTGTAATTGGGGAAAGTAGTT

TTCTCAAATCAATCACTCAGCGTTAGTTTAAAAAAAAGTAATGGCAAAGC

CAAAATTAATTAATCCCCCACTCGCTACATCACCACGAAACAACACGCGT

TCACGCGCGCACACACACACACACAACACGTCCACTTGACGACAAAACAT

TTCTCTCTCTCTCCCTGTCTCTCAACTCACTGAAACTATGCGCCGTCCCA

CATTCCAGCCTGGCTACTACTTATTTGACGCGTTCGTACATACGCCAATT

TTATCTATATATAGAAACTTCACTTCCCATAATAAATAAGCCATCCGTAT

AATAAATAAGCCATCCATATATATATTTCATCGACACTTTTGTGGGTTTG

TCCCCCACAGGCAAACTTAAATGTTTTTCATCACTCCAACTCTCATCCAA

ACACTTGAAACTATCAAACTACTACTACACTAGCACTGATACTGTACTAG

> CsCBF2

TTCATGATGAAAAACGACCTCGGCATCCAATTCAAATCATGGGATGCATA

TCGTTTTTCAAATGGACAATTTTAGGAGTTGTTTGGCAAATATATATATA

TATATATAATTTTATAAATTAAATTATTTAAGTTTATAATCGTATTTGTA

TTTTGTTTTAAAAATTTAAATTCTTTTTGTTGAATATGGCACAGTATAAA

GAAATCATAATATATTTGATATTTATAATTTTTTTCAATAATATTACGGA

TTTGATATTTATAATTTTTTTCAATAATATTACGGCTCCAGATATAAAAA

TTTAAAAATTATTTAGAATTTCACATCAATGGCTCAGACTGATTTCATAT

GTGAACTCCATACTACACTTAGCTTCGTTTGGGAAGTGCGTTAATTTTTT

GACTTTTTTGGTTATTTTAGCCTGGGGGCTAAAAAGCTTGGTTTGGGAAG

AGCTTTTGACTTTTTTGGTTTTTTTTTTTTAATTTTTTGTGAGAATAATG

ATTATGGGTTGGATGTGATATTTTGTCTTATTGGATTAAAAAGTTTATTT

TATTTTTTGTGAATATTTTAGTCAAAAAAGCCTGAAAAACTACTTCCTAA

ACATAGGCTAAAATTTAATTTTCACCATAAATCATTATATCTAAAAAAAC

TGAATAGATATGTCATGATTCATAATATTTTTGTAATTTTTTTGTTTCAC

GCATTTATTTGGAAAAGATAGGTTGCGTTTGGTAGAATTTAAAAATTATG

ATTTGAGAATAGAGTTATATTTTGACTTTATGAATTTTGAAATTATGATT

TGTGCGTGAAAAATTTGTTTGGTTATAAGGCCTCGTTTGGCAAGAGGTTA

TTTTAGCCAATTAGTCAAAAAGTTACTAAATTAGCCAAAAAGCCAACAAC

CCATACAAAAAATTCAAAGATTCTCTTTTCTCTTTACTTTTTGCACATCT

TTTCGTAATTAGCCAAAAAGTAATCTCAAACATACAAGTTTTTTTTACCT

ACCTTTTTAGCCAAATAGCCAAAAAATTACTAAATTAGCCTGTTCCCAAA

CGAGGCCTAAGTTTTAAATTTTTTTTGAATTGATTTGATGTGTATGAGAG

AGAGATTTGTTTGAAAAATTTGTAAACCAAACAAGTTTTGGAAAAAATTT

TTGAAAAAATTTGTTTGATTTAATTACTTTTAAAAAATTATTTATAAAAA

ATTTTTGTTAAAATTTTTTGAGAAACACAAACCAAACAAGGCCAAGATAT

CCCAAACAAGACTTTAATAAAAAATTTAAAACCATTACTCAATAAAAAAA

TGCCAGGCCAAAGTTGCTAGGTGGTGTTTGGGAAGCAAGTTTCCAAATTT

TTTTCTAATTTTTTTCACTAAAAGACAAAAGTTTTACAAAACCAAAAAAA

ACACATCCAAACAAAATATCACATCCAACCCCATAATCATTTCACTTTTT

CTCTCACAAACATCACATCCAATCACATAAAAACTCAACTATCTTTCCAA

ACACTAAAAATTTTTTATCTCATAAAAACCTACATCCAATAAAATCTACA

TAAAAACTCACTTCCCAAACACCATCTATGTCACCCGAAAACCACTATGG

TGTCATACATATATAACCTCACACGCACGCAACACATCAACTTGACCCTT

GACAAAACAGCTGTTCTCTCTCTCTCTCTCTCTCTCTTCCAACTCGTTGC

AAATGTGCTCCGCCCCATCCCCACAGCCTGGCCACTCATCATTTGCCGCG

TTCCCATATGTGCTTATTTGTCTATATATAGTCACTTCACTTCCCATATT

AATAATCTACCCACTTGAACTTCGTTGTTATTCATATTTAAGTACCTTAA

AAAAATACATATTTGATCGTCACTTTGTGGGTTTGCGCCGCAAAAGCAAA

CTTAGGCCTCGTTGCAACTGTGCGCCGCCCCCATCCCCACAGCCTGCTCA

TCACTCTCAACTCTCATCCCAAATCATACTTGTAACCACTACTATACTCT

> CsCBF3

AACCAAAGCACATTTGTTTTTATAAAACTTAACGGTTAACATAACTTTTA

TAAATATTTAAAAACAATTAAGACCTAATCATGAGATTATAAATATCTGA

TTTATTTAGTTTTTTACATTAATAACATTCTTGACAAACTCTACAATATG

AACAGTTTAGATTCTTGACAAACTCTACAATATGAACAGTTTAGATTATC

GAAAAACATTTGAAATGAACTCCCATAAAAGGGCACAATAGGCTATTATG

CCTTGAAGGCACAACAACACCCCAAGTCCCGTCGTATAATACTCCATTGC

TTGGCCTTTTTTATTTTTTGAAAAATTATAAATGCACCTTAAATTTATTT

AAAATATAAATTATTTTTTATGATTTTAAAAATTATATTTCATCTCTCAA

AATTTTAAATTTCATAATAAATAGTTCATTTTATTAATAAATTAAAAAGT

TAATTAATAATTTTTAATGATATCATAAATTTATTTTAATTAATAATTTA

AATAATTTTATTATTGTATAAAAAAACAATTATGAATTTTATTTTTTCAA

TTAATTCTGCTAATTAAAATTTTTTGACAATAAAATATTTATAATTTTTT

AAACTATAGGAGACAATTCTTATTTTAAGCAAACTTTAGGGATATATTTA

TAATTTATCCTTTTTTATTGAAAGATAAATAAAAATATTATATTTTTTAT

CCCTATAGTTTGATTTTACTTTACTCTTTATAATTTCAGTTGAAGTAGTT

TTTTTTTTTTTTTTTTTTTTTTAAAGCATTACTCAGTTTAAATAGTTTAG

TCTATATACTTTAAAACTAGTTCAAATTTGATCAATTCATCTAAATTGAA

AGTTTAAATTTAATAATTTTAAAACACTTATCAAATTTCTAGCCTTTTAA

TAAGTCAAAATGATATTATTAAAAAAAGGAAATAAGACATCGAAGCAACG

CAACTTTGGTATAATCGACGGGAACTCGTCTCTTGAACACATGGACAGAA

TTCCCAATGGAGCAACGTTTGACTCCGACGTCAACAGTCTTCGTATACAT

GAAAAATCAGGAGATGTGTTACGAAGGAGTTATTTAGTGAAAAAATTTAT

GATTTTTAAATATTACTATTTATTATTTCTTTAAAGTATTTTTTTTTTTT

TTCCTAATAATCATTAATGTGTAATTCTCCCTTTCTTATAAAACTCAACG

TAAAATTGGTAAACCTCATTATTTCAATCAATAAATAATAAATTAGAATA

TCGTATAAATTTGTTCTTAAACATTTTAAAAAATTATCAAATATTTACAT

ATAAATATTATATAAAATATTTGATAATTTATTTAATATCCCAAACGTGC

TCTGCAATATTTCTGCGTGAGGGGTGTATTTGGAGAAGGTAGTTTTCTCA

GATGAATGACTTACTTACCAGTACCAGTAATATCATCAAAATAAAAAATA

AAAAAATAATGCCAAGGCAAAAGAATAAGATATTAATCCCCACTTGCTCC

CAAAACCACGTGTTATACATATAACCTATCCACACACACAGACACACAAC

ACATCCTCTCCTCTCCTCACCACAAACCAGCTGTTCTCTCTCTCTATTCC

GAAGTTACGGCACGTCACGTGACCTAATAATGTTATAATGGGTGACAGGA

ATGAAATTTATGAATTTATATTGGATGATTGAAAAATATAAGAAGACTGG

TGCCGCAATAATTTTTCCACTCTCTCCCTCTTTCTTCCTTCCAGCTCACT

GCAGCTGTCCAGCTTGGCTACTCGTCAATTGCCACGCTCACGTATGCGCT

TATTTTATCTATATAAAGTCACTCCACTTCCCATATTAAATAAACTCCAC

TTTGCTGCTTCAAATATATCTGATCCACACATTTTGTGCCCCAAAAGCAA

ATTTAACTGCTTCTCATCACTCCACTCCAACTCTCATCCAAACACTTGAA

ACTATCATACTACAACTACTAGTACATAACCACTGATATTATTGTAGTAT

> CsCBF4

TCGTTCGACCTTCCTTGAAGGCCGGTGAGAATGTTCCTTGGTCCTATCGT

GCTGAGGAGTCGTAGGAATTGTCTTCTGCCCCCCTGCCTGGGCATCTTTC

TTGGCTGCTGGAATCGACGTATCTTGTGACGGGAACGGTGGCTACATGAC

GTTGTTCTGTATCATGAAATTGACAACCGTTTGCAGGTCCGCCAATTTGT

TCGTCATTCATTTCATCTCGTCGTCCCTTCATAAGCATCTTTCTTTCCAT

ACCTGGAGGTCGCCCTTGTTATAATACTTGCTTCGGTCTGATTTCTTCGG

CACGTCGCCCTCCTGGCCGACGTCCTCTTCTCCGTGATTCTTATCACGAG

TTTCTCCGTTGTAGTTGTCTTCTTCGACGCTCAATATGTTTTCCACTTTC

AGTAGAGGTGTCGTCCCCAACCTTCTCTTTCGGTCTGTCGTTGGCCATCT

ATTCTCCTTTCGTTCCCACAGACGGCGTCAAATTGTGTGGATGATTTTCT

GACCACACTGCCAAACAGGTACCCAACTAGAGAGTACCGGCAGAATAGAT

TAAGCCCGAATGACTTTTAACAGAAAAAGAAGATTCTGGCGATGGTTCGT

CCACCCGGGGGCACTTCGACGATCAAGTCAGTACTTAATATCTCTCAAAG

ATAACCAATAGTGCTTAACTCACTAGACAGAATATCAAAAGCATACCATA

GACCCATGGAGGTGCCTTCCTTTTATAGCCAACATAATCCTAATCCCAAT

AGAAGCCTAGTTCTAATGGACTTAGAATTACTGCTAATCTTGGACTCCTA

GTAGGATTAGGATAAACTCTCTAAGAGTCTCATATTTACCCAAACTGAAA

TCTTCTCTCATTCTAGGACTCTTGTCCCTTATTCGTTTATCTCGGGTTCC

GACATATTCACAGATAACCTCGGCTTAGCCGAGATCACCTCGGCATCATG

ACCATGACACGTGGCACCTCGGCTGACACTTTGGTGACACCTCGACCACA

TGTTGTCATTCAAAAATTATGTCCCCACAATATGTATAGTTTTTTAGTTT

TGAGTAAATTTTTTTTGAAAAATTTGGTAACTTTTTTCTTCAGATGTAAA

GAAGTTCTGTTGTACTTTTTAGAAGCCAGCACAATAACAAAGATCTTAAA

AGCAGGCAATCTAATCATCAATTTCTGTGAATGTATACTCGTCGCCGCCT

CACCACCGTTACCCTTTCTATTCGTCTCTTGTCGCCTCTCTTGGTTCATC

TCATTATTGCCTCCCCTCGCCTCACCACCGCCTCGTCACTCCTCGCTTCG

ATAGGAAATTACGTCGACTGCATAGTCCTCTCTGTTCATCGCCTCTTTGT

TCGTCTATTGCAGAGGGGTTAGAGTTCAAAAAACAAAAAGAAACTAAAAA

GTAAAATAAAAAAATATAAAAAAAATTAAAAACGACGTTGTTTGGAAAGG

TTGTCACATAGAACTTCTGAACTCCTTCTAAACTAAGCAAAATGACGTCA

CACGTGGCAGCATTTGAGGTACCAATCAAACGCATCTGGTCACGTCACAT

CTAGCTCCAAAAACACGCACTTTACGATGTTATTAAGACCTAACAAAAAG

GTCCACGTGTTTTCAGCTGCTTCCTACTATCAGTATTTCAAGACTTTGAA

GTTTCGCCACGTCATCACTACCTCAACACGTGTTAAAATTCCACTGACAG

GTTGACTAGAAAGAGTCAAAGATGTTTCCATTTTTGGGAAAAAAAAAAAG

TAAGGGGACCTACTAGGTGGCCGCCCCCAAGCCATCAGCTTCCGTGTTCG

CGTCCTCATCCCAAATACGTGTCCCCCTACCCATCCAGACAATACCAGTA

CTTGCATTTGCTTATATAAAAACCAAATCACAACTCACTTCACTTATAGT

AAGCTGACAAACCACAAAAAGCAAAACCAAATTCGAGTTCAGACCTACTT

GATTATCATTACAAACACTATTTGGATCAAACACAAACACTATCTGGATC

> CsCBF5

TATTTGGTATTTATAATTTTTTCAGTAACATTACGGCTCCAGACATAAAA

ATTTAAAAATGATCTAAAATTTCACATTAATGATTTAGATTGATTTTATA

AGTGAACTCCACATTACACTAATTCAATTTTCACCATAAATTATTATACA

CAGAAAAATTTGAATAGATATGTCATGATTCATAACATTTTTATAATTTT

TTTTGTTTCACGCATGTATTTGGGAAAGATTATTTTCTCAAATCAATCAC

TCACTGATCAATCACATACTCCACAAAAAAAAAAAAAGAAAAAAAAAAGA

AGAAGATAAAAATAAATGTTAAAGACTTCAAAAATTTTATTGAATTATTT

ATCAAATATTTCATATAAATATGAAATCCACACTATATAAATCTCATATA

AATCTCACTCTCATAAAAAATATTTAATAAAAATTTTAAAATCATTAACA

TTACTCAATAAAATAATGCCAAGCCGAGTCCGGATCGTCTGCTGTGATTT

CCTGCGGTGGCTCCTGCGGAGGTCCAAAACGACGTCGTTTTGGACCTTTT

TTTTTTTTTTTCCTTCTCTTTGCTGGTAAGCCTCTTTCTTCCCTTTCATC

TCTCACCCAAACACACAGACGAAGATGGATTCAACTCAGCACCATTAACA

AAAATTGCAAAATTATCTACACCTAGCTCTTCCTCTGTTATCCATCTCTC

TCTCTCTCTCTCACACTCTCTCTCTCTCACACACACACACATATATGTAT

ACATATACACATATATATATATATATGAAATTGTTTTCAAATATTGATAT

GAGTTTGAAATTGTATATGAATCTAAACTTCATAAATCTGATCTTGAAGT

TTTCAAACCCACTTTGTTTTCAAATATATGTATATATCCAAAAAAAAAAA

AAAAAACTTCATGAATGACTTGTATGTTCCAAAAAAAAAAAACACAGATC

ACAAATCAAACCCACTTGCTTTTTTTTTTCGGCAAAGGATCTTTGCCAAT

CTGTTCAAGGCGATGCAGATCGGAACAGATCTGAGACCATTGGAGCAGAT

GAGAGGAAGATGAAGTGAAAGAGAGGCTTAACAACAAAAGAAGAAGAAGA

AGGGGAAAAAAAAAAAAATTGGTCCAAAACGATGTCTCCTTTTTCTTCTC

TTTCATCTCTCACCCAAACACACAGAGATGGAGATGGATGGCCATAGAAT

GGATTTAACTCAGCACCATTAACAAAAATTGCAAAATTATCTACAACTAG

CTCTTCCTCTGTTATCCATATATATCTCTCTCTCTCTCTCTCACACACAC

ACACACATATACATACACTTGATTTGAAAACAAGTTGTAACAGAGGAAGA

ATCAAGTTGTTGAGCACATGTATATATCCAAAAAAAAAAATAAAAATTCA

TGAATGATTTGTATGTTCCAAAAAAAAAACACAGATGATCTTTGCCAAGA

AAAACGCTTGATTTTTTTTTCAAATCAAGTGTTTCAAATCAAGTGTATGT

TTGAAAAAAAACACTTCATGAATCAAACTCACTTATTTTTTTTCAAATCA

AGTGTATGGTTCAAAAAAAATACTTCATGAATCAAACCCACTTGATTTTG

TTGAGCACAAGTGTACGTATACATATCACAGATCAAAACAAATCTGAGAC

TATTGGAGCAGATCAGAGGAAGAGGAAGTGAAAGAGAGGCTTAACAGCAAAGTGAAGGGAAAAAAAAAAAAAATTTCAAAACGACGTCATTTTAAACCGCAACAGGAGCTATCGCAAGAAATCACGGCAGATGATCTGGACTCATCCACT

TGACGCTTGACAAAACAGCTGTTCTCTCTCTCTCTTCCAACTCATTGCAA

CTGAGCGGCGCCCCCATCCCCACAGCCTGGCCCTCTTCATTTGCCGCGTT

CCCATATGTGCTTATTTGTTTATATATAGTCACTTCACTTCCCATGTTAA

ATAATCTACCCACTTTGTTTTTAGACATATTTTATTTACCTTAAATCGAT

> CsCBF6

ATAATGGACATCTACTAGCACCCATAACATTATATGAAAATATTCTATGT

ACATATCTTTTAGAAGCAAAAAAATCTCAATATACACATTTTGAGTAAAA

AAATAAAAATAAATAAATCTTAATTTTTCTATTGTTGTCACTTTGCTTGC

TACACACTCTATACTCTTTTATTGTTTGCAAATCAATATTCTTATAACAA

CTCTTATATTGTTTTAATCTCTAGATTTATACTCTATTAGTGATAAAAAT

ATTAGTTTTTTTATGCATAATTTTTTTTTTCTCAGTCGGATAATCGTACT

GAATTATTTCATTATATTCGGACATCAACTGCCGAAAGTTTTTATATATT

TTTTTTTGGAACAAATTTGTCAAAATAAACACTTTTATTCAGAAGTTAAG

TTTCAAATTAAATGTGTAATTTCGAAAAACTAGTGTCAAAATCTCCAAAC

TGAAAATATATATATATATATATATATATAAAGTGTAGAAATGAGAGTTG

AGTAGTGTGTGTACAATGAGTATGCGTGAAGGGTATAACTGTAACTTTTA

GTGTGTGGATTGAGAAAATTAATTAATAATCAAATTAATAAATGTGTGCA

TTGAGAGAATATATACATTGAGGTAATTAAGTGTGGAAATAGAATTTCTC

CAAAAATATGTTGTCCATAAGGACATGCTAAGATAAAATAACCAAATTAC

CCCATTGTTTGAACAATTGTTAATGTTGGTGTTCTTTTGAGTCGTGGTGA

GAAATGGAGGGTTTTAGAGAAGAAAGTACAGAGAAAAATGGCCACAATAG

AGAAATCCTCTGCCAGTGCCTTCTCTTTCTTTCTTTATTTTTTTTTTTTT

TTTTTTATTTTTAAATATTTTGAAATTTTGCCTTTGGGGCACCGCAAGAA

CCACTGCAGGGGAGCACGATAGAGGATTGAGACTCCAACAATAACTAACA

AATAACAAATATAGAGGGTGTTTGGGATGTTGGTTATTTAGTAAATTATT

TTAATTTTTGACTATTTTGGTAAGTTAAAATAGTTGTGTTTAGGAAGATG

ATTGAATTTTTTTTGTAATTAGATATGATATTTGTAAGAAAAAAAATAAA

ATAATTATAAATTTGATATAATATTTTATTTTATTAATTATTTTAATCTA

TTTTATTTTTCAGTTGTCAAAAAAAAAAAGGAAAAAAAAAATCTATTGTC

AAGCACCCCATAATATAAATAAATAATTCAAACAACGTCCCTAATTTTCA

ACGTGCCCAAGACAAACAGTTTCCGTCACGTGTTCTTTGCGCGTCTTTTG

CGCGTGTCTATCTCATTCACGTACGTCCTTACTCAGACCCTTGAGATTCC

TATTTATAACTGTTACTTATTTATTTTATTTTTTTAACTGTCCGCATGTA

GACACTTAAAAAGAGAAAAGTTCGACGCCATCAAATTTTTATTTTGAAAA

TAATTAAGAAAGATTTATGATTTTTGCATTATCTTGATTAGTGTGTAATA

TTTTATTCTAAGTATAATCTAAAAATTATAAATTTTTTATGATTATTTTT

ATAAATTTTTTTTCATAATACCTAATATTACTGCACTTAAAAAGACCCAT

TTCTCACTCACTTCTGTCAACTTTCTGTCATTACAAGTATGCATTATATA

TAGTAAAACCAAGGCACACTTTCTATATACTGTGATAAGAGTCTTTTTCA

GCCTACCAAATCTTGGTTTTAAGTCTCATTTACATCTTTATATAATTTTG

TGTTTTAAATATAAAAAGATAAATCACTTTATTTATTTTTGGACACTTTT

AACCTTTGCTTTATATAACCTTTGCTTTATATATAGTAGAGATTTAGAAA

ACTAAGCACACTATGCAAATGAAATTGTCAATACATTAAGATTGTTGGAG

AGAGAGGGAGAGAGAGTTTGTCTGTGTGTAGTGGGCAACAACAATATATT

TGTGTGAAATTCTATCATCTAAGTGAGTTTTGTGTGTAATAGAATAGATA

tgt
